# Phosphodiesterase 2A inhibition corrects the aberrant behavioral traits observed in genetic and environmental preclinical models of Autism Spectrum Disorder

**DOI:** 10.1038/s41398-022-01885-2

**Published:** 2022-03-25

**Authors:** Sara Schiavi, Emilia Carbone, Francesca Melancia, Alessandra di Masi, Marielle Jarjat, Fréderic Brau, Silvia Cardarelli, Mauro Giorgi, Barbara Bardoni, Viviana Trezza

**Affiliations:** 1 Deptartment of Science, University “Roma Tre”, Rome, Italy; 2 Université Côte d’Azur, CNRS, IPMC, 06560 Valbonne, France; 3 Deptartment of Biology and Biotechnology “Charles Darwin”, Sapienza University of Rome, 00185 Rome, Italy; 4 Université Côte d’Azur, Inserm, CNRS, IPMC, 06560 Valbonne, France

**Keywords:** Neuroscience, Learning and memory

## Abstract

Pharmacological inhibition of phosphodiesterase 2A (PDE2A), which catalyzes the hydrolysis of cyclic adenosine monophosphate (cAMP) and cyclic guanosine monophosphate (cGMP), has recently been proposed as a novel therapeutic tool for Fragile X Syndrome (FXS), the leading monogenic cause of Autism Spectrum Disorder (ASD). Here, we investigated the role of PDE2A in ASD pathogenesis using two rat models that reflect one of either the genetic or environmental factors involved in the human disease: the genetic *Fmr1-*^*Δ*^*exon 8* rat model and the environmental rat model based on prenatal exposure to valproic acid (VPA, 500 mg/kg). Prior to behavioral testing, the offspring was treated with the PDE2A inhibitor BAY607550 (0.05 mg/kg at infancy, 0.1 mg/kg at adolescence and adulthood). Socio-communicative symptoms were assessed in both models through the ultrasonic vocalization test at infancy and three-chamber test at adolescence and adulthood, while cognitive impairments were assessed by the novel object recognition test in *Fmr1-*^*Δ*^*exon 8* rats (adolescence and adulthood) and by the inhibitory avoidance test in VPA-exposed rats (adulthood). PDE2A enzymatic activity in VPA-exposed infant rats was also assessed. In line with the increased PDE2A enzymatic activity previously observed in the brain of *Fmr1*-KO animals, we found an altered upstream regulation of PDE2A activity in the brain of VPA-exposed rats at an early developmental age (*p* < 0.05). Pharmacological inhibition of PDE2A normalized the communicative (*p* < 0.01, *p* < 0.05), social (*p* < 0.001, *p* < 0.05), and cognitive impairment (*p* < 0.001) displayed by both *Fmr1-*^*Δ*^*exon 8* and VPA-exposed rats. Altogether, these data highlight a key role of PDE2A in brain development and point to PDE2A inhibition as a promising pharmacological approach for the deficits common to both FXS and ASD.

## Introduction

The term Autism Spectrum Disorder (ASD) refers to a group of persistent developmental psychiatric disorders emerging in the early life and characterized by impairments in social interaction, restricted communication abilities, and stereotyped/repetitive behaviors. Common co-morbid features include anxiety and intellectual disability [1]. To date, no treatment is available for ASD, leading to the use of off-label medications to attenuate the patients’ symptoms [1].

Although the pathogenesis of ASD is still debated, different factors (*e.g*., rare gene mutations, gene variations, and adverse environmental events) have been suggested to interact each other in complex ways thus affecting early brain development and contributing to the risk of ASD [2]. Several environmental factors have been identified as prenatal (*e.g*., maternal age, exposure to medications, tobacco, toxins or viruses) as well as natal (*e.g*., complications during the parturition such as hypoxia) risks for ASD [3]. Exposure to harmful environmental factors can change the expression of developmental key genes in critical periods of embryo formation and increase the risk of genomic imprinting diseases such as ASD. One of the best examples of prenatal environmental factors involved in the pathogenesis of ASD is maternal exposure to valproic acid (VPA), a widely used and effective antiepileptic and mood stabilizer drug [4]. On the basis of the clinical association between maternal use of VPA and increased risk of neurodevelopmental delay, cognitive deficits and autism in children [4, 5], prenatal VPA exposure in rodents has been validated as an animal model recapitulating the main behavioral and neural features of ASD [6–9]. On the other side, increasing evidence points to a strong genetic contribution to ASD and new technologies now allow for the identification of many mutations affecting single genes. Thus, genetic alterations identified in ASD are fast expanding. According to the SFARI database (https://gene.sfari.org/database/human-gene/), more than 1000 genes have been implicated in ASD, with both de novo and rare inherited variants and including single-gene disorders as well as copy number variations [10]. The monogenic leading form of ASD is Fragile X Syndrome (FXS), the most common form of inherited intellectual disability (ID) with an estimated prevalence of 1:4000 males and 1:7000 females [11, 12]. Indeed, besides ID, FXS patients show several ASD-like symptoms, including social dysfunction, hyperactivity, stereotypic movements, hand flapping and hand biting, speech delay, and a relative lack of expressive language ability. Overall, approximately 30% of patients with FXS meet the full diagnostic criteria for ASD [13], while over 90% of individuals with FXS display some ASD symptoms [14]. FXS is caused by the absence of expression of the *FMR1* gene, which ultimately leads to the lack of the encoded Fragile X Mental Retardation Protein (FMRP), a modulator of translation of synaptic proteins and of mRNA transport at the synapse. FMRP plays a key role in several neural pathways that are disrupted in ASD [15], including cAMP and cGMP signaling. In particular, recent studies pointed to phosphodiesterases (PDEs) as potential therapeutic targets for FXS [16–21]. The main function of the PDEs is to catalyze the hydrolysis of cyclic adenosine monophosphate (cAMP) and cyclic guanosine monophosphate (cGMP), two essential secondary messengers that modulate a wide array of intracellular processes related to neurobehavioral functions, including memory and cognition [22]. Interestingly, there is genetic evidence for a role of PDEs in ASD [23, 24]. PDEs such as PDE2A, PDE4D, and PDE10A have been associated with multiple autism-like behaviors and cognitive deficit at different ages in mouse models [19, 20, 25, 26]. Accordingly, some PDE inhibitors (PDE2A, PDE3, PDE4/4D, and PDE10A) have been proposed to treat neurodevelopmental diseases, including ASD and FXS [21, 22, 27]. We recently found that FMRP negatively regulates the translation of *Pde2a* mRNA in mouse cortex and hippocampus. Consequently, PDE2A levels and activity are increased in *Fmr1*-KO mice, resulting in reduced levels of cAMP and cGMP [19]. We also showed that pharmacological inhibition of PDE2A rescued the alteration in morphology of dendritic spines, exaggerated mGluR-dependent long-term depression (LTD) and the deficits in social and communicative domains displayed by both *Fmr1*-KO mice and rats [20].

On this basis, to investigate whether pharmacological inhibition of PDE2A corrects the core and co-morbid features of ASD, we performed a longitudinal study from infancy through adulthood in two animal models that reflect two of the most common genetic and environmental factors involved in the pathogenesis of ASD, namely the genetic *Fmr1-*^*Δ*^*exon 8* rat model [28] and the environmental model based on prenatal VPA exposure in rats [8]. In particular, socio-communicative symptoms were assessed in both models through the ultrasonic vocalization (USV) test at infancy and three-chamber test at adolescence and adulthood, while cognitive impairments were assessed by the novel object recognition test in *Fmr1-*^*Δ*^*exon 8* rats at adolescence and adulthood and by the inhibitory avoidance test in VPA-exposed rats at adulthood. This type of approach allows to monitor drug effects in the course of the animals’ development since both *Fmr1-*^*Δ*^*exon 8* and VPA-exposed rats present core and co-morbid features of ASD from infancy to adulthood [7, 28, 29], showing age-specific behavioral alterations. Moreover, since we previously observed an increased PDE2A enzymatic activity in the brain of *Fmr1*-KO animals [20], we measured PDE2A enzymatic activity in the brain of VPA-exposed infant rats showing that is elevated during early post-natal brain development.

## Materials and methods

### Animals

We used two rat models of ASD to recapitulate either genetic [1] or environmental [2] factors known to be involved in the human disease:

1. *Fmr1-*^*Δ*^*exon 8* rats on a Sprague-Dawley background (Horizon Discovery, formerly SAGE Labs, USA), proposed as genetic model of ASD and rat model of FXS [28]; the corresponding wild-type (WT) animals were used as controls.

2. Wistar rats (Charles River, Italy) born from dams treated with VPA (500 mg/kg) on gestational day (GD) 12.5 [29]; Wistar rats born from dams treated with saline solution (SAL) on GD 12.5 were used as controls.

**Fig. 1:**
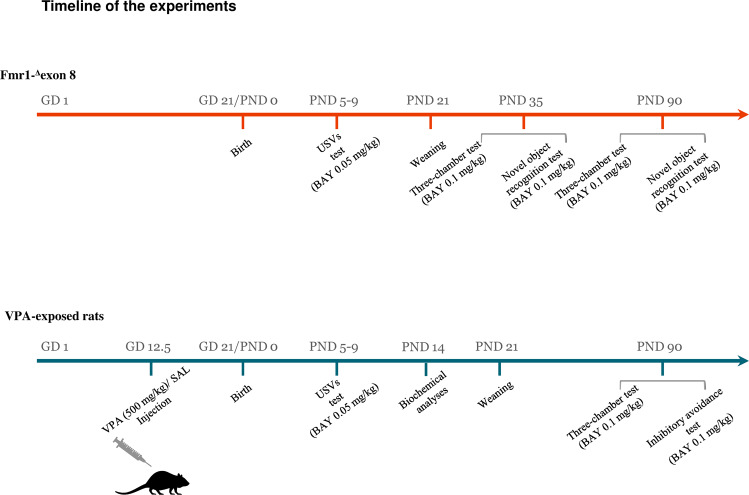
Timeline of the experiments. Sequence of the behavioral and biochemical experiments performed in the two rat models of ASD.

Pregnant rats from both models were individually housed in Macrolon cages (40 (l) × 26 (w) × 20 (h) cm), under controlled conditions (temperature 20–21 °C, 55–65% relative humidity and 12/12 h light cycle with lights on at 07:00 h). Newborn litters found up to 17:00 h were considered to be born on that day (postnatal day (PND) 0). On PND 1, the litters were culled to eight animals (six males and two females) to reduce any litter size-induced variability in the growth and development of pups during the postnatal period. On PND 21, the pups were weaned and housed in groups of three (same sex and same genotype or environmental manipulation). The male offspring from both rat models were tested during development. Except for the USVs analysis, in which the same animals were used at PNDs 5 and 9, different animals were used for each behavioral test during adolescence and adulthood. The biochemical analyses were performed on VPA-exposed rats at PND 14 in animals belonging to a different cohort compared to the ones used for behavioral testing. *Fmr1-*^*Δ*^*exon 8* offspring were genotyped between PND 35 and PND 90 (see Fig. 1 for the timeline of the experiments).

One pup per litter from different litters per treatment group was randomly used in each experiment. Sample size was based on our previous experiments and power analysis performed with the software GPower. Sample size (*n*) is indicated in the figure legends. Potential outliers within each data set were calculated using the GraphPad Prism 8 software (Grubbs’ method).

Behavioral testing was performed between 9:00 a.m. and 3:30 p.m. during the 12-h light period; brain samples were collected in the same time interval.

A trained observer who was unaware of the treatments scored the behavioral tests using the Observer 3.0 software (Noldus, The Netherlands). The experiments were performed in agreement with the ARRIVE (Animals in Research: Reporting In Vivo Experiments) guidelines [30], the guidelines of the Italian Ministry of Health (D.L. 26/14) and the European Community Directive 2010/63/EU.

### Drug treatment

VPA (Cayman Chemical, USA) was dissolved in SAL at the concentration of 250 mg/ml and administered at a dose (500 mg/kg) and time (GD 12.5) that have been shown to induce autistic-like behavioral changes in the offspring [7, 29].

BAY607550 (BAY) (Cayman Chemical, USA) was dissolved in a solution containing 10% DMSO, 8.75% Tween 80, 8.75% polyethylene glycol and saline (i.e., VEH) [20]. BAY or VEH were administered intraperitoneally (i.p.) 30 min before testing at the dose of 0.05 mg/kg at infancy and 0.1 mg/kg at adolescence and adulthood [20]. In the inhibitory avoidance test only, BAY was administered immediately after the acquisition trial to exclude any drug-induced variability in the training phase (e.g., changes in pain sensitivity, motivation, and locomotion). Solutions were administered in a volume of 2.5 ml/kg at infancy, 2 ml/kg at adolescence and 1 ml/kg at adulthood.

### Behavioral tests

#### Isolation-induced ultrasonic vocalizations

On PNDs 5 and 9, the isolation-induced ultrasonic vocalizations (USVs) emitted by each pup removed from the nest and placed into a Plexiglas arena were detected for 3 min by an ultrasound microphone (Avisoft Bioacoustics, Germany) sensitive to frequencies between 10 and 200 kHz [7]. The USVs were analyzed quantitatively using Avisoft Recorder software (version 5.1).

#### Three-chamber test

The test was performed as previously described [7]. *Fmr1-*^*Δ*^*exon 8* rats were tested at PND 35 and PND 90 while VPA-exposed rats were tested at PND 90. The apparatus was a rectangular three-chamber box with two lateral chambers (30 (l) × 35 (w) × 35 (h) cm) connected to a central chamber (15 (l) × 35 (w) × 35 (h) cm). Each lateral chamber contained a small Plexiglas cylindrical cage. Each experimental rat was individually allowed to explore the apparatus for 10 min and then confined in the central compartment. An unfamiliar stimulus animal was confined into a cage located in one chamber of the apparatus, while the cage in the other chamber was left empty. Both doors to the side chambers were then opened, allowing the experimental animal to explore the apparatus for 10 min. The percentage of time spent in social approach (sniffing the stimulus animal) and the percentage of time spent exploring the empty chamber were scored using the Observer 3.0 software (Noldus, The Netherlands).

#### Novel object recognition test

*Fmr1-*^*Δ*^*exon 8* rats were tested in the novel object recognition test at PND 35 and PND 90. The experimental arena was 40 cm wide × 40 cm deep × 40 cm high, with the floor covered with sawdust. The arena was positioned in a dimly illuminated room (from 2 to 5 lux). On the training trial, each rat was individually placed into an open-field arena containing two identical objects (i.e., A1 and A2) equidistant from each other, and allowed to explore the objects for 5 min. Thirty minutes later, one copy of the familiar object (i.e., A3) and a new object (i.e., B) were placed in the same location as during the training trial. Each rat was placed in the apparatus for 5 minutes, and the time spent exploring each object was recorded. The discrimination index was calculated as the difference in time exploring the novel and the familiar objects, expressed as the percentage ratio of the total time spent exploring both objects [31].

#### Inhibitory avoidance

On the first day, 90-day-old rats were individually placed in the illuminated compartment of an inhibitory avoidance apparatus (Ugo Basile, Italy). After 10 s, the sliding door was opened, and the time taken by the animal to enter the dark compartment was measured (latency). Once the animal entered the dark compartment, the sliding door was closed and a mild shock (0.6 mA) was delivered through the floor for 2 s. Twenty-four hours later, the animal was placed in the lit compartment and the latency to re-enter (retention latency) into the dark compartment was recorded [32].

### Biochemical analyses

#### PDE2A activity assay

Brains were homogenized without cerebellum using a glass homogenizer (15 strokes, 4 °C) in 20 mM Tris-HCl buffer pH 7.2 containing 0.2 mM EGTA, 5 mM β-mercaptoethanol, 2% (*v*/*v*) antiprotease cocktail (Merck KGaA, Germany), 1 mM PMSF, 5 mM MgCl_2_, 0.1% (*v*/*v*) Triton X-100. The homogenates were centrifuged at 14,000 × *g* for 30 min at 4 °C and the pellet was resuspended in a final volume of 0.15 ml of a buffer composed of 60 mM Hepes pH 7.2, 0.1 mM EGTA, 5 mM MgCl_2_, 0.5 mg/ml bovine serum albumin (BSA), and 30 mg/ml soybean trypsin inhibitor. The measure the PDE activity was measured according to the method reported by Thompson and Appleman [33]. The reaction was started by adding triturated substrates at a final concentration of 1 μM [^3^H] cGMP. The reaction was stopped by adding 50 μl of 0.1 N HCl and then neutralized with 50 μl of 0.1 N NaOH in 0.1 M Tris-HCl pH 8.0. Subsequently, 25 μl of 2 mg/ml of 5ʹ-nucleotidase (snake venom from Crotalus atrox; Merck KGaA) in 0.1 M Tris-HCl pH 8.0 were added. Samples were gently mixed and incubated at 30°C for 30 min to allow complete conversion of 5’-nucleotide to its corresponding nucleoside. Unhydrolyzed cyclic nucleotide and the corresponding nucleoside were separated by DEAE-Sephadex A-25 columns. The eluate was mixed with ULTIMA GOLD scintillation liquid (PerkinElmer, USA) and counted on a Tri-Carb 2100TR Liquid Scintillation Counter (2000CA; Packard Instruments, USA). To evaluate the enzymatic activity of PDE2A, the specific inhibitor BAY was added to the reaction mix at a final concentration of 0.1 μM.

#### RNA preparation and RT-qPCR

Total RNA derived from frozen tissues was extracted by RNeasy Kits (Qiagen, Germany) according to the manufacturer’s instructions. For the quantification of the mRNA level of *Pde2a*, RNA was retrotranscribed with Superscript IV reverse transcriptase (Invitrogen, USA). Quantitative PCR (RT-qPCR) was performed by a Light Cycler 480 (Roche, Swiss) using the MasterMix SYBRGreen (Roche, Swiss) following the manufacturer’s instructions and according to the MIQE guidelines [34]. The TBP transcript was used for normalization. The relative expression of the transcripts was quantified using the 2^−ΔΔ^ Ct method [35]. Rat primer sequences to amplify *Pde2A* and *Tbp* were: PDE2A-FW: 5ʹ-CAGTCGAGCCACTGACCA-3ʹ; PDE2A-RV: 5ʹ-ATCGCACGTTCATCCTCAT-3ʹ; TBP-FW: 5ʹ-CCCACCAGCAGTTCAGTAGC-3ʹ; TBP- RV: 5ʹ-CAATTCTGGGTTTGATCATTCTG-3ʹ.

#### Immunofluorescence

Brain obtained from PND14 VPA and control rats were fixed in 4% (*w*/*v*) paraformaldehyde for 48 h. Brain slices of 50 µm were obtained by vibratome (Microm HM650V – Thermo Scientific^TM,^, Illkirch, France) and Immunofluorescence was performed as described [36], using polyclonal anti-PDE2A primary antibody (# PD2A-101AP, 1:200, FabGennix, Frisco, TX 7) followed by a treatment with 70% True Black (Biotium, USA) diluted in ethanol. After washing, slices were incubated with the goat anti-rabbit Alexa 488 secondary antibody (1:1000; Invitrogen Italy). DAPI (5 µg/ml; Invitrogen, USA) was used to counterstain nuclei. Mosaic images were collected using a FV10i laser scanning confocal microscope (Olympus, Japan) equipped with a UPLSAP 60X/1.35NA objective. The ImageJ software (http://imagej.nih.gov/ij/) was used for image analysis. Fluorescence intensity quantification was performed using a home-made ImageJ program [37, 38]. Cortex, hippocampal CA3 and CA1 regions were manually selected on the whole brain images. In those brain regions, DAPI nuclei staining was used to define segmented regions of interest for each cell. Then, the A488 PDE2A mean fluorescence intensity was measured on each of these cellular regions of interest (through a local maximal detection). In Supplementary Fig. 1 we show an example of segmentation in order to measure the fluorescence level for each cell. One slide for each animal was analyzed. In total we analyzed the following number of slides: CA3: 10 SAL, 10 VPA; CA1: 7 SAL, 10 VPA; cortex: 8 SAL, 8 VPA. In Supplementary Tables 1–6, mean of the fluorescence intensity/per cell (Mean) and the area of each cell analyzed (Area) are reported.

The code of the macro used is available in the Supplementary Material section.

### Statistical analysis

Behavioral data are expressed as mean ± standard error (SEM) while biochemical data represent the mean ± SEM of 3 brains for each experimental group analyzed in duplicate.

All the behavioral data, except the ones obtained by the inhibitory avoidance test, were analyzed by two-way ANOVA, with either genotype (WT or *Fmr1-*^*Δ*^*exon8*) or prenatal treatment (VPA or SAL), and treatment (BAY or VEH) as factors. Repeated measures ANOVA with time, pretreatment and treatment as factors was used to analyze the data from the inhibitory avoidance test. The Student–Newman–Keuls post hoc test was used for individual group comparisons. To measure PDE2A activity with or without BAY in the brains of SAL- and VPA-exposed animals, Kruskal–Wallis analysis followed by Dunn’s multiple comparisons post hoc test was used. To assess the impact of the prenatal treatment (VPA or SAL) on PDE2A activity and expression, data were analyzed with unpaired Student’s *t*-test. The accepted value for significance was set at *p* < 0.05. The software GraphPad Prism 8 (GraphPad Software, USA) was used for statistical analysis of the data. Potential outliers within each data set were calculated using the Grubbs’ method available in the GraphPad software (USA).

## Results

### Pharmacological inhibition of PDE2A normalizes the communicative and social deficits displayed by *Fmr1-*^*Δ*^*exon 8* rats in the course of development

**Fig. 2:**
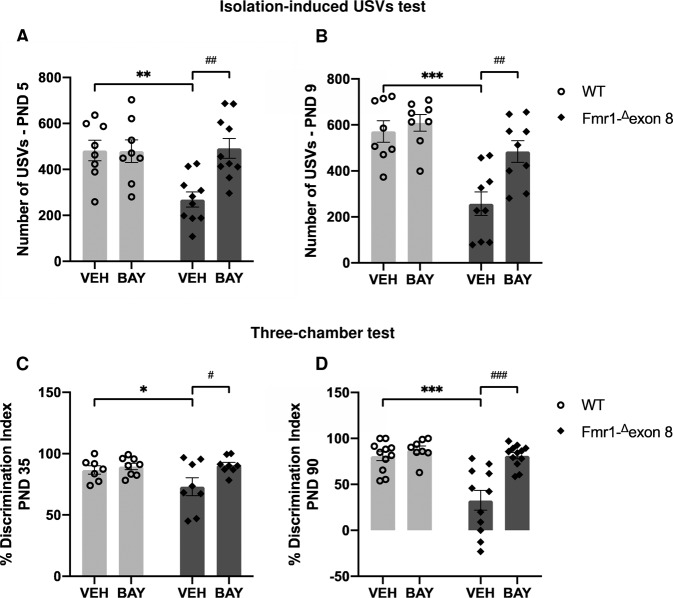
Pharmacological inhibition of PDE2A normalizes the communicative and social deficits displayed by *Fmr1-*^*Δ*^*exon 8* rats in the course of development. *Fmr1-*^*Δ*^*exon 8* rats emit less USVs when removed from the nest at PNDs 5 (**A**) and 9 (**B**), and this communicative deficit is reversed upon BAY607550 injection (PND 5: WT-VEH, *n* = 8; WT-BAY, *n* = 8; KO-VEH, *n* = 10; KO-BAY, *n* = 10; PND 9: WT-VEH, *n* = 8; WT-BAY, *n* = 8; KO-VEH, *n* = 9; KO-BAY, *n* = 9). *Fmr1-*^*Δ*^*exon 8* juvenile and adult rats display reduced sociability in the three-chamber test, as they show a lower discrimination index (**C**, **D**). At both ages, the altered phenotype displayed by *Fmr1-*^*Δ*^*exon 8* rats is rescued by PDE2A inhibition (PND 35: WT-VEH, *n* = 7; WT-BAY, *n* = 8; KO-VEH, n = 8; KO-BAY, *n* = 8; PND 90: WT-VEH, *n* = 11; WT-BAY, *n* = 8; KO-VEH, *n* = 11; KO-BAY, *n* = 12). Data represent mean ± S.E.M. *p < 0.05, **p < 0.01, ***p < 0.001 vs WT-VEH group, ^#^*p* < 0.05, ^##^*p* < 0.01, ^###^*p* < 0.001 vs *Fmr1-*^*Δ*^*exon 8*-VEH group (Student’s–Newman–Keuls post hoc test).

*Fmr1-*^*Δ*^*exon 8* rat pups showed a marked reduction in USV emission when compared to WT animals at both PNDs 5 (*p* < 0.01, Fig. 2A) and 9 (*p* < 0.001, Fig. 2B). As expected, acute (i.p.) administration of BAY 30 min before testing normalized the altered USV emission displayed by *Fmr1-*^*Δ*^*exon 8* pups at both PNDs 5 and 9 (PND 5: F_(genotype) 1,32_ = 5.668, *p* < 0.05; F_(treatment) 1,32_ = 6.764, *p* < 0.05; F_(genotype x treatment) 1,32_ = 7.122, *p* < 0.05); PND 9 (F_(genotype) 1,30_ = 22.72, *p* < 0.001; F_(treatment) 1,30_ = 8.266, *p* < 0.01; F_(genotype × treatment) 1,30_ = 4.280, *p* < 0.05). These results confirm that PDE2A blockade rescues the communicative deficit displayed by *Fmr1-*^*Δ*^*exon 8* rat pups (PND 5: *p* < 0.01; PND 9: *p* < 0.01) [20].

We have recently reported that *Fmr1-*^*Δ*^*exon 8* juvenile and adult rats show reduced sociability in the three-chamber test [28]. Here, we confirm and extend these previous findings, as both juvenile (PND 35: *p* < 0.05, Fig. 2C) and adult (PND 90: *p* < 0.001, Fig. 2D) *Fmr1-*^*Δ*^*exon 8* rats showed a lower discrimination index compared to WT animals. At both ages, the altered phenotype displayed by *Fmr1-*^*Δ*^*exon 8* rats in the three-chamber test was rescued by PDE2A inhibition (PND 35: F_(genotype) 1,27_ = 2.038, *p* = n.s.; F_(treatment) 1,27_ = 5.139, *p* < 0.05; F_(genotype × treatment) 1,27_ = 2.692, *p* = n.s.; PND 90: F_(genotype) 1,38_ = 16.23, *p* < 0.001; F_(treatment) 1,38_ = 16.67, *p* < 0.001; F_(genotype × treatment) 1,38_ = 9.445, *p* < 0.01). Indeed, the lower discrimination index displayed by *Fmr1-*^*Δ*^*exon 8* rats was normalized after treatment with BAY (PND 35: *p* < 0.05; PND 90: *p* < 0.001).

### Pharmacological inhibition of PDE2A normalizes the cognitive deficits displayed by *Fmr1-*^*Δ*^*exon 8* rats in the course of development

**Fig. 3:**
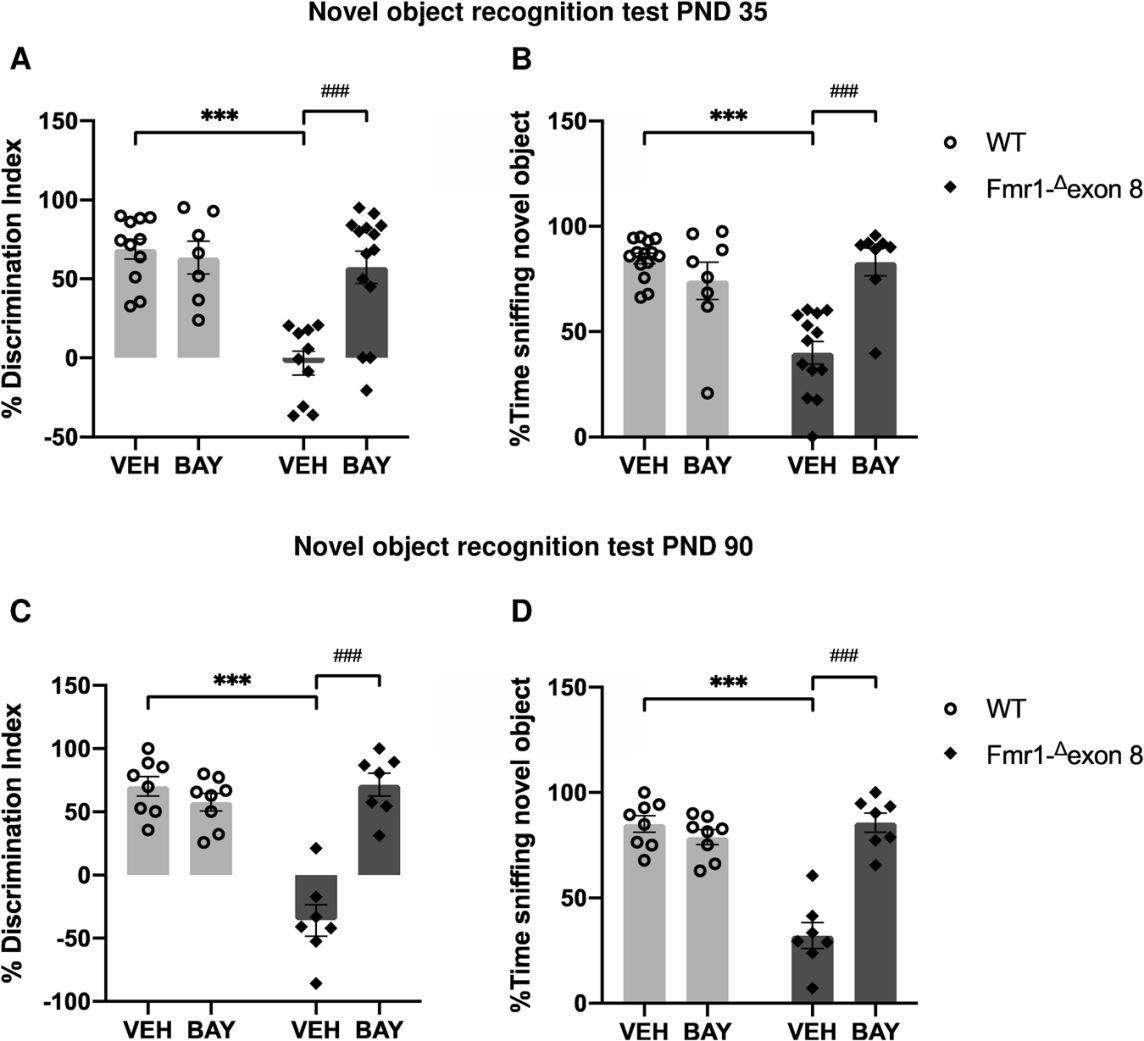
Pharmacological inhibition of PDE2A normalizes the cognitive deficits displayed by *Fmr1-*^*Δ*^*exon 8* rats in the course of development. Juvenile and adult *Fmr1-*^*Δ*^*exon 8* rats show a reduced discrimination index (**A**, **C**) and spend less time sniffing the novel object (**B**, **D**) in the novel object recognition test. Both these parameters are normalized when *Fmr1-*^*Δ*^*exon 8* rats are treated with BAY607550 (PND 35: WT-VEH, *n* = 11; WT-BAY, *n* = 7; KO-VEH, *n* = 10; KO-BAY, *n* = 14; PND 90: WT-VEH, *n* = 8; WT-BAY, *n* = 8; KO-VEH, *n* = 7; KO-BAY, *n* = 7). Data represent mean ± S.E.M. ***p < 0.001 vs WT-VEH group, ^###^*p* < 0.001 vs *Fmr1-*^*Δ*^*exon 8*-VEH group (Student’s–Newman–Keuls post hoc test).

In line with our previous findings [28], *Fmr1-*^*Δ*^*exon 8* rats displayed impaired object recognition both at PND 35 (*p* < 0.001, Fig. 3A and B) and PND 90 (*p* < 0.001, Fig. 3C and D). These deficits in object recognition were normalized when both juvenile and adult *Fmr1-*^*Δ*^*exon 8* rats were treated with BAY (PND 35: % Discrimination index (F_(genotype) 1,38_ = 17.68, *p* < 0.001; F_(treatment) 1,38_ = 8.827, *p* < 0.01; F_(genotype x treatment) 1,38_ = 12.59, *p* < 0.01); % Time sniffing novel object (F_(genotype) 1,38_ = 10.08, *p* < 0.01; F_(treatment) 1,38_ = 8.363, *p* < 0.01; F_(genotype x treatment) 1,38_ = 22.67, *p* < 0.001); PND 90: % Discrimination index (F_(genotype) 1,26_ = 25.45, *p* < 0.001; F_(treatment) 1,26_ = 26.89, *p* < 0.001; F_(genotype x treatment) 1,26_ = 42.94, *p* < 0.001); % Time sniffing novel object (F_(genotype) 1,26_ = 25.45, *p* < 0.001; F_(treatment) 1,26_ = 26.89, *p* < 0.001; F_(genotype x treatment) 1,26_ = 42.94, *p* < 0.001). Thus, PDE2A inhibition normalizes the cognitive deficits displayed by *Fmr1-*^*Δ*^*exon 8* rats in the course of development (PND 35: *p* < 0.001; PND 90: *p* < 0.001).

### PDE2A activity in the brain of VPA-exposed rats

**Fig. 4:**
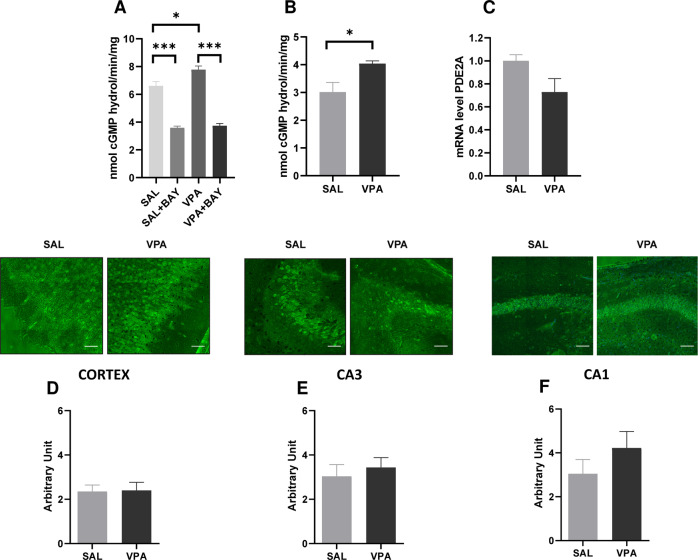
PDE2A activity, but not expression, is increased in the brain of VPA-exposed rats. **A** cGMP degradation level was measured in forebrain from VPA and control rats (SAL) at PND 14. Levels of cGMP degradation catalyzed by total extracts in the absence or presence of BAY607550 are shown (Kruskal–Wallis analysis followed by Dunn’s multiple comparisons test; SAL *n* = 3; SAL + BAY *n* = 3; VPA *n* = 3; VPA + BAY *n* = 3). **B** PDE2A-specific cGMP degradation measured as the difference of values obtained from forebrain extracts in the absence and presence of BAY607550 (unpaired Student’s *t*-test, SAL *n* = 3; VPA *n* = 3). **C** At PND 14, mRNA was purified from forebrain of VPA- and SAL-exposed rats. *Pde2a* mRNA expression level was quantified by RT-qPCR and compared between the two samples (unpaired Student’s *t*-test, SAL *n* = 4; VPA *n* = 4) *p < 0.05; ***p < 0.001. **D**–**F** PDE2A expression was quantified by immunofluorescence on brain slices obtained from VPA- and SAL-exposed rats at PND 14. The intensity of immunofluorescence was measured in **D** cortex, **E** hippocampal CA3 and **F** CA1 regions. In upper panels (**D**–**F**), an example of PDE2A immunofluorescence in the corresponding brain region in SAL- and VPA-treated rats is shown. The intensity of immunofluorescence is indicated as arbitrary unit (A.U.). In lower panels (**D**–**F**), each graph shows the measure of the intensity of the fluorescence *per* cell in SAL- and VPA-treated rats. Data represent mean ± S.E.M of the immunofluorescence/cell. Unpaired Student’s *t*-test. No significant difference was measured. Cortex: 1202 cells analyzed from 8 SAL-treated rat brains and 970 cells analyzed from 8 VPA-treated rat brains; CA3 hippocampal regions: 541 cells analyzed from 8 SAL-treated rat brains and 790 analyzed from 8 VPA-treated rat brains; CA1: 398 cells analyzed from 7 SAL-treated rat brains and 899 cell analyzed from 10 VPA-treated rat brains.

We measured the total amount of hydrolyzed cGMP in whole brain extracts from control and VPA-exposed rats at PND 14, finding an increased cGMP degradation in VPA animals compared to SAL controls (F_(3,8)_ = 82.69; *p* < 0.001_;_ Fig. 4A). However, when the PDE2A inhibitor BAY607550 was added to the reaction mix, no difference was observed between the SAL + BAY and VPA + BAY groups, suggesting that about 50% of the total amount of hydrolyzed cGMP is PDE2A-dependent. Thus, we measured the PDE2A-dependent hydrolysis of cGMP [33] in whole brain extracts from control and VPA-exposed rats at PND 14. We observed that hydrolyzed cGMP was higher in VPA-exposed animals compared to SAL-exposed controls (*t* = 2.91; *p* < 0.05; df = 4; Fig. 4B), suggesting an increased activity of PDE2A in the brain of VPA-exposed rats. However, at the same age the expression levels of *Pde2a* mRNA measured by RT-qPCR did not display a significative difference in the whole brain of VPA- and SAL-exposed rats (*t* = 2.12; *p* = n.s.; df = 6; Fig. 4C). Moreover, the quantitative measure of PDE2A protein expression levels assayed by immunofluorescence did not show any difference between SAL- and VPA-exposed animals in cortex, CA3 and CA1 hippocampal regions (Fig. 4D–F). These results suggest an altered regulation of the activity of PDE2A by upstream pathways rather than an elevated protein level.

### Pharmacological inhibition of PDE2A normalizes the communicative, social, and cognitive deficits displayed by VPA-exposed rats

**Fig. 5:**
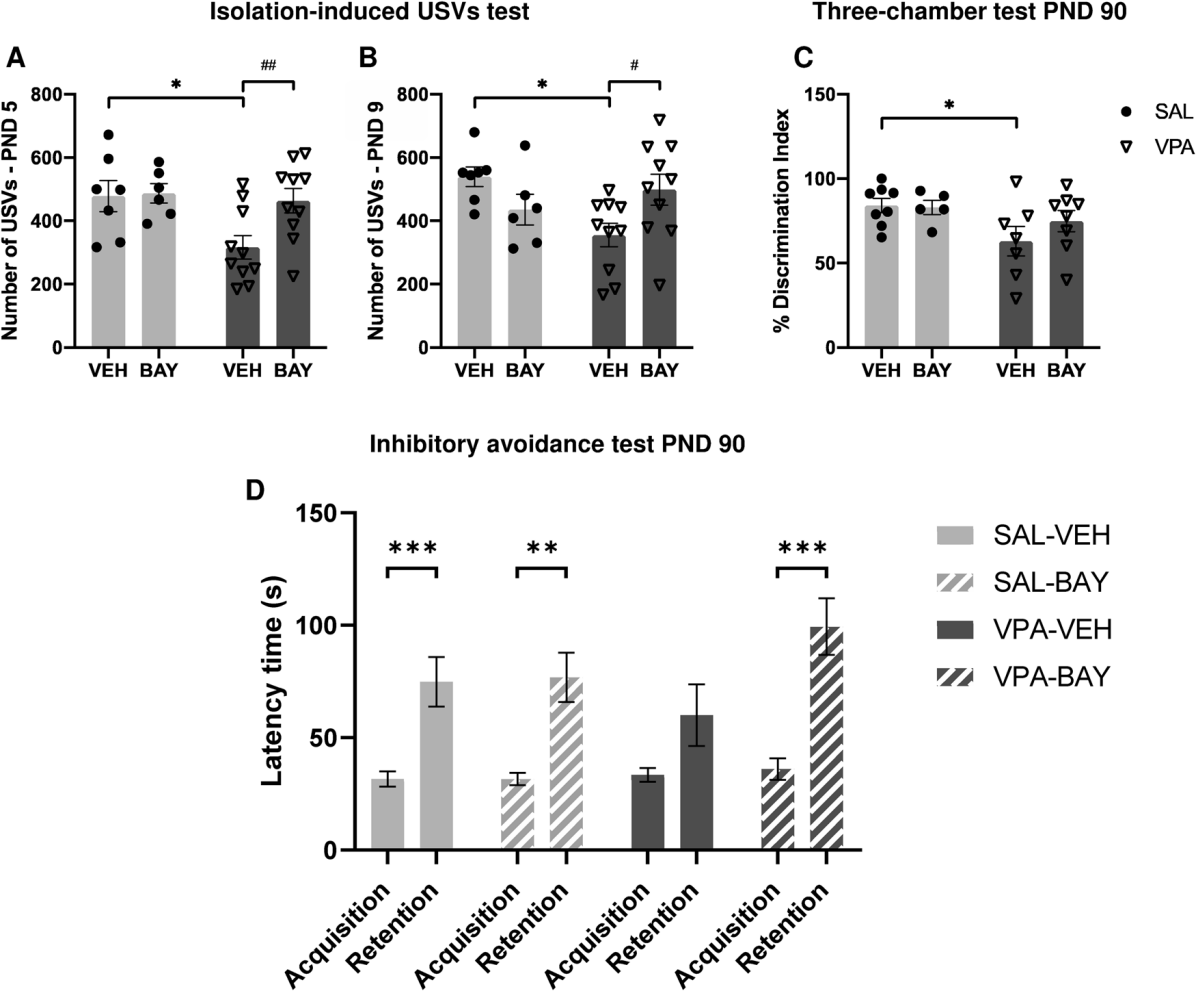
Pharmacological inhibition of PDE2A normalizes the communicative, social and cognitive deficits displayed by VPA-exposed rats. PDE2A inhibition by BAY607550 normalizes the altered USV profile displayed by VPA-exposed pups both PNDs 5 (**A**) and 9 (**B**) (PND 5: SAL-VEH, *n* = 7; SAL-BAY, *n* = 6; VPA-VEH, *n* = 10; VPA-BAY, *n* = 10, PND 9: SAL-VEH, *n* = 7; SAL-BAY, *n* = 6; VPA-VEH, *n* = 10; VPA-BAY, *n* = 10). Moreover, administration of BAY607550 rescues the lower discrimination index displayed by VPA-exposed rats in the three-chamber test (**C**) (SAL-VEH, *n* = 8; SAL-BAY, *n* = 5; VPA-VEH, *n* = 7; VPA-BAY, *n* = 8). At adulthood, VPA-exposed rats present impaired consolidation of aversive memories, that is reversed following administration of BAY607550 (**D**) (SAL-VEH, *n* = 31; SAL-BAY, *n* = 30; VPA-VEH, *n* = 16; VPA-BAY, *n* = 16). Data represent mean ± S.E.M. *p < 0.05 vs SAL-VEH group, ^#^*p* < 0.05, ^##^*p* < 0.01 vs *Fmr1-*^*Δ*^*exon 8*-VEH group (Student’s–Newman–Keuls post hoc test); **p < 0.01, ***p < 0.001 vs acquisition time (Student’s–Newman–Keuls post hoc test).

In line with our previous findings [7, 29], VPA-exposed pups displayed communication deficits at infancy as they emitted a reduced number of USVs compared to the SAL-exposed pups (PND 5: *p* < 0.05, Fig. 5A; PND 9: *p* < 0.05, Fig. 5B). Pharmacological inhibition of PDE2A rescued the altered USV profile displayed by VPA-exposed pups at both PND 5 (F_(pre-treatment) 1,29_ = 5.002, *p* < 0.05; F_(treatment) 1,29_ = 3.541, *p* = n.s.; F_(pre-treatment × treatment) 1,29_ = 2.792, *p* = n.s.) and PND 9 (F_(pre-treatment) 1,29_ = 1.849, *p* = n.s.; F_(treatment) 1,29_ = 0.1972, *p* = n.s.; F_(pre-treatment × treatment) 1,29_ = 7.754, *p* < 0.01), since VPA-exposed pups treated with BAY emitted an increased number of USVs compared to VPA-exposed pups treated with vehicle (PND 5: *p* < 0.01; PND 9: *p* < 0.05).

At adulthood, VPA-exposed rats showed altered sociability in the three-chamber test, as they spent less time exploring the social stimulus compared to the SAL-exposed rats (*p* < 0.05, Fig. 5C). PDE2A blockade normalized the reduced sociability of VPA-exposed rats in the three-chamber apparatus (% Discrimination index (F_(pre-treatment) 1,24_ = 5.108, *p* < 0.05; F_(treatment) 1,24_ = 0.6829, *p* = n.s.; F_(pre-treatment x treatment) 1,24_ = 1.024, *p* = n.s.). Indeed, no differences were found between VPA-exposed rats treated with BAY and SAL-exposed rats treated with vehicle (*p* = n.s).

We have previously shown that adult rats prenatally exposed to VPA display an altered consolidation of aversive memories in the inhibitory avoidance test, while they show intact object recognition [29]. Here, we show that inhibition of PDE2A activity through administration of BAY rescued the impaired emotional memory displayed by VPA-exposed rats in the inhibitory avoidance test: (F_(time) 1,178_ = 44.44, *p* < 0.001; F_(pretreatment) 1,178_ = 0.373, *p* = n.s.; F_(treatment) 1,178_ = 3.045, *p* = n.s.; F_(time × pretreatment) 1,178_ = 0.000, *p* = n.s; F_(time × treatment) 1,178_ = 1.917, *p* = n.s.; F_(pretreatment × treatment) 1,178_ = 2.559, *p* = n.s.; F_(time × pretreatment × treatment) 1,178_ = 1.513, *p* = n.s. Fig. 5D). Particularly, good memory retention was observed in animals prenatally exposed to SAL and treated with either VEH (*p* < 0.001) or BAY (*p* < 0.01), but not in VPA-exposed animals (*p* = n.s.). This altered phenotype was rescued when VPA-exposed rats were treated with BAY (*p* < 0.001).

## Discussion

PDE2A is a dual substrate enzyme that catalyzes the hydrolysis of both cAMP and cGMP. It is expressed in both the periphery and in the central nervous system (CNS) with high expression in the brain [22]. In particular, it is mainly localized in the cortex, hippocampus and striatum [22, 39, 40] suggesting that PDE2A may regulate neuronal cAMP and cGMP levels in brain areas involved in emotions, perception, learning, and memory [41], brain functions often disrupted in patients affected by FXS or other forms of ASD [42].

We recently found that FMRP, whose functional absence causes FXS, binds the mRNA coding for PDE2A in the mouse cortex and hippocampus and negatively modulates its synaptic expression [19, 20]. Consequently, the loss of FMRP results into elevated PDE2A expression and enzymatic activity, leading to decreased levels of cAMP and cGMP [20]. Remarkably, PDE2A inhibition by acute and chronic administration of BAY607550 reverted the socio-cognitive deficits, the aberrant maturation of dendritic spines and the exaggerated hippocampal mGluR-dependent LTD displayed by *Fmr1*-KO mice [20].

On this basis, as both genetic and environmental animal models of ASD are essential to prove the validity of new therapeutic targets, we wondered whether a deregulation of PDE2A activity might also contribute to the altered behavioral phenotype observed in a widely used environmental animal model of ASD based on prenatal VPA exposure. To be consistent with our previous studies, we measured PDE2A activity at PND 14, when the synaptogenesis peaks in the brain cortex and hippocampus and when FMRP is most highly expressed, [19, 20, 43–45] in line with its important role in both brain development and synaptic activity. At the molecular level, we found that the total amount of hydrolyzed cGMP was higher in VPA-exposed animals compared to controls, while no difference was observed in the presence of BAY607550, indicating that about 50% of the total amount of hydrolyzed cGMP is PDE2A-dependent in VPA-exposed animals during synaptogenesis. This confirms and reinforces the critical role of PDE2A for proper development of brain and behavior. Furthermore, at the same age no differences were found between VPA- and SAL-exposed animals in whole brain *Pde2a* mRNA levels and in PDE2A expression in various brain regions. These results lead to hypothesize an altered regulation of the enzymatic activity of PDE2A by upstream pathways instead of an increased level of the protein, as we previously observed in *Fmr1*-KO mouse cortex and hippocampus [20]. An up-regulation of intracellular nitric oxide (NO) was detected in neuronal cells differentiated from adipose tissue-derived stem cells treated with VPA [46]. cGMP is formed in response to NO by NO-sensitive guanylyl cyclases [47] and, importantly, cGMP activates PDE2A inducing the degradation of both cGMP and cAMP [22]. Considering the high expression of PDE2A in the brain [40] and its high activity during development (our data), its deregulation may have a strong impact on neuronal functioning.

The increased activity of PDE2A in the brain of VPA-exposed rats at PND 14 prompted us to test the hypothesis that PDE2A inhibition through administration of BAY607550 would correct the aberrant behavioral traits displayed by VPA-exposed animals. Social and communicative impairments in the VPA rodent model of ASD have been long documented [7, 8, 29, 48]. Interestingly, we found that inhibition of PDE2A activity rescued the social communication deficits displayed by VPA-exposed rats by normalizing the reduced number of USV emitted by these animals at PND 5 and 9. Furthermore, in line with our previous results [20, 28], we found quantitative reduction in the USVs emitted by *Fmr1-*^*Δ*^*exon 8* rats when separated from their mother and siblings compared with control animals, both at PNDs 5 and 9. The socio-communicative deficits displayed by *Fmr1-*^*Δ*^*exon 8* and VPA-exposed pups were long-lasting. Indeed, in line with previous studies [28], *Fmr1-*^*Δ*^*exon 8* animals showed reduced sociability in the three-chamber test both at adolescence and adulthood, similarly to VPA-exposed rats. Interestingly, pharmacological inhibition of PDE2A normalized the performance of *Fmr1-*^*Δ*^*exon 8* and VPA-exposed rats in the three-chamber test. The positive effects of PDE2A blockade on the socio-communicative domain of these rats could be explained by PDE2A expression in a specific class of neurons in the olfactory bulb, which plays a prominent role in rodent social recognition [49]. Even if further studies are needed to confirm that modulation of PDE2A activity in neurons of the olfactory bulb may influence social behavior, we can underline that additional indirect evidence supports the hypothesis that PDE2A plays a role in sociality [22, 50]. Indeed, patients with bipolar disorder, schizophrenia and major depressive disorder showed altered expression of brain PDE2A [22]. Furthermore, PDE2A inhibition ameliorated social withdrawal in a rat model of schizophrenia [50]. The modulation of cAMP and cGMP has been also reported to be key in learning and memory. Indeed, mutations of *PDE2A* have been found associated with intellectual disability [22, 51, 52] and PDE2A seems to have a prominent role in memory disorders [53–55]. Several studies have shown that *Fmr1*-KO mice display cognitive impairments [56–59]. More recently, we have shown that *Fmr1-*^*Δ*^*exon 8* rats display impaired object recognition memory both at young age and adulthood, being unable to discriminate between a familiar and a novel object [28]. Here, we show that the PDE2A inhibitor BAY607550 rescued the impaired object recognition displayed by both juvenile and adult *Fmr1-*^*Δ*^*exon 8* rats. In line with our findings, BAY607550 was effective in improving memory acquisition and consolidation in object recognition tasks [60–62] and in reversing stress-induced cognitive impairment [63] in rats.

Cognitive dysfunction is a frequent comorbid feature in ASD [64]. Although some authors reported abnormally high and longer lasting fear responses in VPA-exposed animals [65], we have previously shown that adult VPA-exposed rats display an altered consolidation of aversive memories in the inhibitory avoidance test, while they show intact object recognition [29]. Remarkably, here we show that inhibition of PDE2A activity through administration of BAY607550 rescued the impaired emotional memory displayed by VPA-exposed rats in the inhibitory avoidance test.

In conclusion, our results show that PDE2A enzymatic activity was increased in the brain of VPA-exposed rat during synaptogenesis. PDE2A inhibition by using BAY607550 normalized the altered communicative, social, and cognitive phenotype of VPA-exposed rats. This finding is in line with previous studies demonstrating a role of other PDE enzymes in the VPA model of ASD. As an example, PDE10A inhibition corrected the altered behavioral repertoire displayed by VPA-exposed rats by altering protein markers associated with neuronal survival, neurogenesis, neuronal transcription factor, neuronal transmission, neuronal inflammation, and neuronal oxidative stress [25]. Like PDE2A, both PDE5 and PDE6 are activated by cGMP and, remarkably, PDE5 inhibition was proven to protect from VPA-induced teratogenesis [66]. Converging on the same pathway, these two studies suggest a key role of cGMP level (or the cGMP/cAMP ratio) in the pathogenesis of VPA-induced autism, although further studies are needed to confirm this possibility. Along the same line, inhibition of PDE4 and PDE4D has been shown to ameliorate the altered phenotype observed in animal models of FXS and in patients affected by this disorder [16, 21].

Collectively, our findings are suggestive of a shared aberrant mechanism involving PDE2A in both genetic and environmental animal models of ASD. Thus, PDE2A inhibition may be a promising pharmacological target for early correction of autistic-like traits common to both ASD and FXS.

## Supplementary information

Supplementary info macro

Supplementary figure 1

Supplementary Table 1

Supplementary Table 2

Supplementary Table 3

Supplementary Table 4

Supplementary Table 5

Supplementary Table 6
