# Supplementary material for: Phosphodiesterase 2A inhibition corrects the aberrant behavioral traits observed in genetic and environmental preclinical models of Autism Spectrum Disorder
Source: Transl Psychiatry. 2022 Mar 25;12:119. doi: 10.1038/s41398-022-01885-2 (PMC8956682; doi:10.1038/s41398-022-01885-2)
Supplement: Supplementary file 1 — Supplementary info macro [file 41398_2022_1885_MOESM1_ESM.docx]

**Supplementary code**: macro_PDE2A

// @boolean(label="Summary dans le tableau de resultats", value=false) summarize

function close_all_Images_apart(keepthisimageopen){

for (i=nImages; i>=1; i--) {

selectImage(i);

nom=getTitle();

if (nom!=keepthisimageopen) close();

}

}

function close_Image(image){

selectWindow(image);

close();

}

function Summary_to_Result() {

// close("Results");

selectWindow("Summary");

IJ.renameResults("Summary","Results");

}

function Concatenate_Summary_to_Results() {

setResult("Label", nResults, "Summary");

selectWindow("Summary");

text = getInfo("window.contents");

lines = split(text, "\n");

labels = split(lines[0], "\t");

for (i=1; i<lines.length; i++) {

items=split(lines[i], "\t");

setResult("Label", i-1, items[0]);

for (j=1; j<labels.length; j++) setResult(labels[j], nResults-i, items[j]);

}

updateResults();

}

// Initialisation ----------------------------------------

run("Colors...", "foreground=white background=black selection=yellow");

run("Set Measurements...", "area mean integrated limit redirect=None decimal=2");

run("Options...", "iterations=1 count=1 black");

run("Clear Results");

Fichier=getTitle();

fin=indexOf(Fichier, ".oif");

Fichier_trunc=substring(Fichier,0, fin-1);

run("Split Channels");

// suppression du canal de la transmission

for (i=nImages; i>=1; i--) {

selectImage(i);

nom=getTitle();

if (matches(nom, ".*C3.*")) close();

if (matches(nom, ".*C1.*")) {

if (nSlices>1) {

run("Z Project...", "projection=[Sum Slices]");

close_Image("C1-"+Fichier);

}

rename("Noyaux");

}

if (matches(nom, ".*C2.*")) {

if (nSlices>1) {

run("Z Project...", "projection=[Sum Slices]");

close_Image("C2-"+Fichier);

}

rename("Neurones");

}

}

// Selection de la zone des neurones

setTool("freeline");

selectWindow("Neurones");

resetMinAndMax();

waitForUser("Tracez une ligne epaisse sur les neurones et cliquez sur OK");

run("Line to Area");

selectWindow("Noyaux");

run("Restore Selection");

for (i=nImages; i>=1; i--) {

selectImage(i);

run("Crop");

}

for (i=nImages; i>=1; i--) {

selectImage(i);

run("Select None");

}

// Segmentation des regions des noyaux par Find Max

selectWindow("Noyaux");

run("Mean...", "radius=4");

resetMinAndMax();

run("Find Maxima...", "prominence=170 output=[Segmented Particles]");

run("16-bit");

run("Multiply...", "value=257");

// Combinaison des regions des noyaux avec les neurones et mesures

imageCalculator("AND create", "Noyaux Segmented","Neurones");

rename(Fichier);

run("Invert LUT");

run("Restore Selection");

run("Clear Outside");

run("Set Measurements...", "area mean integrated limit display redirect=None decimal=2");

setThreshold(1, 65535);

run("Analyze Particles...", "size=10-Infinity show=Outlines display clear summarize");

rename("Drawing of Result of Noyaux Segmented");

// Mise en forme des resultats

selectWindow("Drawing of Result of Noyaux Segmented");

run("Invert");

run("Merge Channels...", "c1=[Drawing of Result of Noyaux Segmented] c2=Neurones c3=Noyaux");

close_all_Images_apart("RGB");

rename(Fichier);

if (summarize==true) Concatenate_Summary_to_Results();

close("Summary");

//run("Summarize");

/* Macro automatique d'analyse du marquage PDE2 sur coupes de cerveau

En entree : dossier / images oif de mosaiques: Dapi, PDE2 vert, transmission

Masque sur image noyaux applique image PDE2

Mesure d'instensite

F. Brau pour Marielle Jarjat Equipe Bardoni ImageJ 1.52o Juillet 2019

*/
