# Supplementary material for: Phosphodiesterase 2A inhibition corrects the aberrant behavioral traits observed in genetic and environmental preclinical models of Autism Spectrum Disorder
Source: Transl Psychiatry. 2022 Mar 25;12:119. doi: 10.1038/s41398-022-01885-2 (PMC8956682; doi:10.1038/s41398-022-01885-2)
Supplement: Supplementary file 2 — Supplementary figure 1 [file 41398_2022_1885_MOESM2_ESM.pdf]

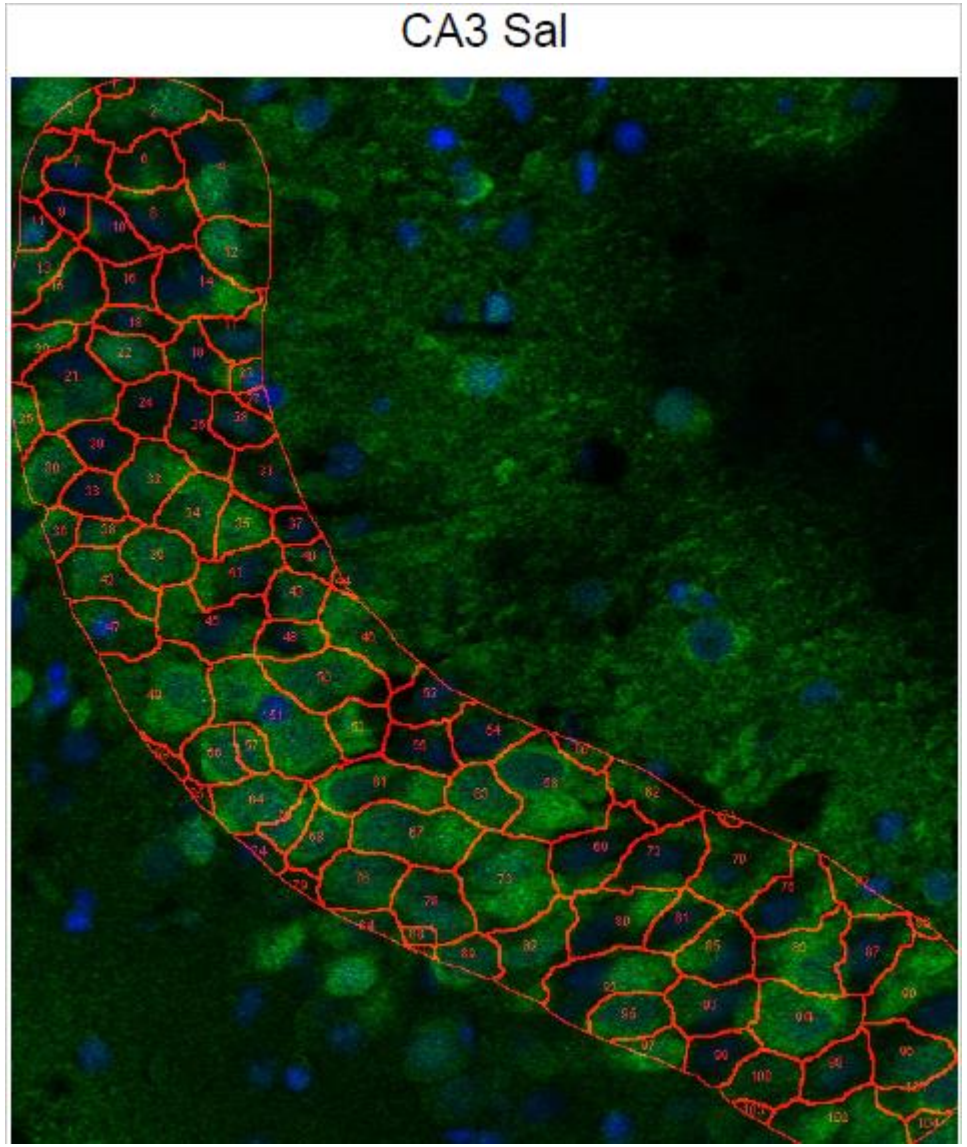

Supplementary Figure 1: an example of segmentation in order to measure the fluorescence level for each cell
