# Supplementary material for: Phosphodiesterase 2A inhibition corrects the aberrant behavioral traits observed in genetic and environmental preclinical models of Autism Spectrum Disorder
Source: Transl Psychiatry. 2022 Mar 25;12:119. doi: 10.1038/s41398-022-01885-2 (PMC8956682; doi:10.1038/s41398-022-01885-2)
Supplement: Supplementary file 5 — Supplementary Table 3 [file 41398_2022_1885_MOESM5_ESM.pdf]

Supplementary Table 3: Measurees of PDE2A expression by immunofluorescence in CA1

Image	Label	Area
201953	Rat_Brain_SAL15.3_PDE2_CA1_Merge_2.oif	89.09334574381523
201953	Rat_Brain_SAL15.3_PDE2_CA1_Merge_2.oif	176.1267297363284
201953	Rat_Brain_SAL15.3_PDE2_CA1_Merge_2.oif	126.1726572672528
201953	Rat_Brain_SAL15.3_PDE2_CA1_Merge_2.oif	129.94925381130662
201953	Rat_Brain_SAL15.3_PDE2_CA1_Merge_2.oif	712.2317755126963
201953	Rat_Brain_SAL15.3_PDE2_CA1_Merge_2.oif	447.5266904703783
201953	Rat_Brain_SAL15.3_PDE2_CA1_Merge_2.oif	260.7568250189891
201953	Rat_Brain_SAL15.3_PDE2_CA1_Merge_2.oif	282.72975036621136
201953	Rat_Brain_SAL15.3_PDE2_CA1_Merge_2.oif	268.13835462782157
201953	Rat_Brain_SAL15.3_PDE2_CA1_Merge_2.oif	199.64462639702722
201953	Rat_Brain_SAL15.3_PDE2_CA1_Merge_2.oif	26.436175808376777
201953	Rat_Brain_SAL15.3_PDE2_CA1_Merge_2.oif	369.248143920899
201953	Rat_Brain_SAL15.3_PDE2_CA1_Merge_2.oif	164.62527662489174
201953	Rat_Brain_SAL15.3_PDE2_CA1_Merge_2.oif	168.57353664822074
201953	Rat_Brain_SAL15.3_PDE2_CA1_Merge_2.oif	219.90091695149775
201953	Rat_Brain_SAL15.3_PDE2_CA1_Merge_2.oif	13.73307834201391
201953	Rat_Brain_SAL15.3_PDE2_CA1_Merge_2.oif	171.14848883734834
201953	Rat_Brain_SAL15.3_PDE2_CA1_Merge_2.oif	69.18038214789507
201953	Rat_Brain_SAL15.3_PDE2_CA1_Merge_2.oif	60.94053514268672
201953	Rat_Brain_SAL15.3_PDE2_CA1_Merge_2.oif	158.10206441243514
201953	Rat_Brain_SAL15.3_PDE2_CA1_Merge_2.oif	43.43086025661899
201953	Rat_Brain_SAL15.3_PDE2_CA1_Merge_2.oif	503.48898471408495
201953	Rat_Brain_SAL15.3_PDE2_CA1_Merge_2.oif	162.56531487358964
201953	Rat_Brain_SAL15.3_PDE2_CA1_Merge_2.oif	560.4812598334427
201953	Rat_Brain_SAL15.3_PDE2_CA1_Merge_2.oif	58.880573391384644
201953	Rat_Brain_SAL15.3_PDE2_CA1_Merge_2.oif	155.87043918185788
201953	Rat_Brain_SAL15.3_PDE2_CA1_Merge_2.oif	14.248068779839432
201953	Rat_Brain_SAL15.3_PDE2_CA1_Merge_2.oif	455.2515470377611
201953	Rat_Brain_SAL15.3_PDE2_CA1_Merge_2.oif	160.33368964301238
201953	Rat_Brain_SAL15.3_PDE2_CA1_Merge_2.oif	212.17606038411492
201953	Rat_Brain_SAL15.3_PDE2_CA1_Merge_2.oif	18.882982720269126
201953	Rat_Brain_SAL15.3_PDE2_CA1_Merge_2.oif	158.78871832953584
201953	Rat_Brain_SAL15.3_PDE2_CA1_Merge_2.oif	38.45261935763895
201953	Rat_Brain_SAL15.3_PDE2_CA1_Merge_2.oif	264.7050850423181
201953	Rat_Brain_SAL15.3_PDE2_CA1_Merge_2.oif	998.3947954644113
201953	Rat_Brain_SAL15.3_PDE2_CA1_Merge_2.oif	209.6011081949873
201953	Rat_Brain_SAL15.3_PDE2_CA1_Merge_2.oif	442.54844957139824
201953	Rat_Brain_SAL15.3_PDE2_CA1_Merge_2.oif	25.23453145345056
201953	Rat_Brain_SAL15.3_PDE2_CA1_Merge_2.oif	201.7045881483293
201953	Rat_Brain_SAL15.3_PDE2_CA1_Merge_2.oif	297.14948262532596
201953	Rat_Brain_SAL15.3_PDE2_CA1_Merge_2.oif	81.71181613498277
201953	Rat_Brain_SAL15.3_PDE2_CA1_Merge_2.oif	78.96520046657997
201953	Rat_Brain_SAL15.3_PDE2_CA1_Merge_2.oif	534.3884109836163
201953	Rat_Brain_SAL15.3_PDE2_CA1_Merge_2.oif	73.30030565049925
201953	Rat_Brain_SAL15.3_PDE2_CA1_Merge_2.oif	421.2621781412767
201953	Rat_Brain_SAL15.3_PDE2_CA1_Merge_2.oif	214.236022135417
201953	Rat_Brain_SAL15.3_PDE2_CA1_Merge_2.oif	144.5406495496964
201953	Rat_Brain_SAL15.3_PDE2_CA1_Merge_2.oif	212.51938734266528

201954	Rat_Brain_SAL1.2_PDE2_CA1_Merge.oif	146.94393825954884
201954	Rat_Brain_SAL1.2_PDE2_CA1_Merge.oif	409.4173980712897
201954	Rat_Brain_SAL1.2_PDE2_CA1_Merge.oif	263.6751041666671
201954	Rat_Brain_SAL1.2_PDE2_CA1_Merge.oif	528.3801892089851
201954	Rat_Brain_SAL1.2_PDE2_CA1_Merge.oif	946.3807612440336
201954	Rat_Brain_SAL1.2_PDE2_CA1_Merge.oif	389.5044344753695
201954	Rat_Brain_SAL1.2_PDE2_CA1_Merge.oif	10.299808756510432
201954	Rat_Brain_SAL1.2_PDE2_CA1_Merge.oif	243.41881361219654
201954	Rat_Brain_SAL1.2_PDE2_CA1_Merge.oif	77.59189263237859
201954	Rat_Brain_SAL1.2_PDE2_CA1_Merge.oif	129.94925381130662
201954	Rat_Brain_SAL1.2_PDE2_CA1_Merge.oif	302.47105048285636
201954	Rat_Brain_SAL1.2_PDE2_CA1_Merge.oif	183.3365958658857
201954	Rat_Brain_SAL1.2_PDE2_CA1_Merge.oif	324.2723123508035
201954	Rat_Brain_SAL1.2_PDE2_CA1_Merge.oif	137.3307834201391
201954	Rat_Brain_SAL1.2_PDE2_CA1_Merge.oif	637.7298255072709
201954	Rat_Brain_SAL1.2_PDE2_CA1_Merge.oif	13.218087904188389
201954	Rat_Brain_SAL1.2_PDE2_CA1_Merge.oif	370.27812479655
201954	Rat_Brain_SAL1.2_PDE2_CA1_Merge.oif	65.74711256239159
201954	Rat_Brain_SAL1.2_PDE2_CA1_Merge.oif	256.46523803710977
201954	Rat_Brain_SAL1.2_PDE2_CA1_Merge.oif	84.45843180338555
201954	Rat_Brain_SAL1.2_PDE2_CA1_Merge.oif	157.24374701605927
201954	Rat_Brain_SAL1.2_PDE2_CA1_Merge.oif	133.89751383463562
201954	Rat_Brain_SAL1.2_PDE2_CA1_Merge.oif	121.36607984754792
201954	Rat_Brain_SAL1.2_PDE2_CA1_Merge.oif	225.56581176757845
201954	Rat_Brain_SAL1.2_PDE2_CA1_Merge.oif	256.12191107855944
201954	Rat_Brain_SAL1.2_PDE2_CA1_Merge.oif	284.6180486382383
201954	Rat_Brain_SAL1.2_PDE2_CA1_Merge.oif	402.3791954210076
201954	Rat_Brain_SAL1.2_PDE2_CA1_Merge.oif	97.16152926974841
201954	Rat_Brain_SAL1.2_PDE2_CA1_Merge.oif	427.0987364366326
201954	Rat_Brain_SAL1.2_PDE2_CA1_Merge.oif	519.4536882866761
201954	Rat_Brain_SAL1.2_PDE2_CA1_Merge.oif	221.10256130642398
201954	Rat_Brain_SAL1.2_PDE2_CA1_Merge.oif	254.2336128065325
201954	Rat_Brain_SAL1.2_PDE2_CA1_Merge.oif	139.04741821289085
201954	Rat_Brain_SAL1.2_PDE2_CA1_Merge.oif	247.88206407335107
201954	Rat_Brain_SAL1.2_PDE2_CA1_Merge.oif	191.0614524332685
201954	Rat_Brain_SAL1.2_PDE2_CA1_Merge.oif	96.81820231119806
201954	Rat_Brain_SAL1.2_PDE2_CA1_Merge.oif	138.18910081651495
201954	Rat_Brain_SAL1.2_PDE2_CA1_Merge.oif	88.40669182671454
201954	Rat_Brain_SAL1.2_PDE2_CA1_Merge.oif	146.08562086317295
201954	Rat_Brain_SAL1.2_PDE2_CA1_Merge.oif	165.48359402126763
201954	Rat_Brain_SAL1.2_PDE2_CA1_Merge.oif	255.26359368218354
201954	Rat_Brain_SAL1.2_PDE2_CA1_Merge.oif	59.39556382921016
201954	Rat_Brain_SAL1.2_PDE2_CA1_Merge.oif	197.41300116644996
201954	Rat_Brain_SAL1.2_PDE2_CA1_Merge.oif	263.6751041666671
201954	Rat_Brain_SAL1.2_PDE2_CA1_Merge.oif	174.23843146430147
201954	Rat_Brain_SAL1.2_PDE2_CA1_Merge.oif	126.51598422580314
201954	Rat_Brain_SAL1.2_PDE2_CA1_Merge.oif	123.76936855740036
201954	Rat_Brain_SAL1.2_PDE2_CA1_Merge.oif	144.5406495496964
201954	Rat_Brain_SAL1.2_PDE2_CA1_Merge.oif	279.12481730143276
201954	Rat_Brain_SAL1.2_PDE2_CA1_Merge.oif	12.188107028537345

201955	Rat_Brain_SAL10.2_PDE2_CA1_2_Merge.oif	22.65957926432295
201955	Rat_Brain_SAL10.2_PDE2_CA1_2_Merge.oif	147.8022556559247
201955	Rat_Brain_SAL10.2_PDE2_CA1_2_Merge.oif	357.91835428873753
201955	Rat_Brain_SAL10.2_PDE2_CA1_2_Merge.oif	25.921185370551253
201955	Rat_Brain_SAL10.2_PDE2_CA1_2_Merge.oif	83.60011440700967
201955	Rat_Brain_SAL10.2_PDE2_CA1_2_Merge.oif	125.4860033501521
201955	Rat_Brain_SAL10.2_PDE2_CA1_2_Merge.oif	268.31001810709677
201955	Rat_Brain_SAL10.2_PDE2_CA1_2_Merge.oif	15.964703572591171
201955	Rat_Brain_SAL10.2_PDE2_CA1_2_Merge.oif	209.42944471571212
201955	Rat_Brain_SAL10.2_PDE2_CA1_2_Merge.oif	402.03586846245724
201955	Rat_Brain_SAL10.2_PDE2_CA1_2_Merge.oif	83.4284509277345
201955	Rat_Brain_SAL10.2_PDE2_CA1_2_Merge.oif	163.0803053114152
201955	Rat_Brain_SAL10.2_PDE2_CA1_2_Merge.oif	758.0659244791678
201955	Rat_Brain_SAL10.2_PDE2_CA1_2_Merge.oif	237.75391879611584
201955	Rat_Brain_SAL10.2_PDE2_CA1_2_Merge.oif	89.43667270236558
201955	Rat_Brain_SAL10.2_PDE2_CA1_2_Merge.oif	287.87965474446656
201955	Rat_Brain_SAL10.2_PDE2_CA1_2_Merge.oif	313.2858496771923
201955	Rat_Brain_SAL10.2_PDE2_CA1_2_Merge.oif	62.82883341471364
201955	Rat_Brain_SAL10.2_PDE2_CA1_2_Merge.oif	112.09625196668854
201955	Rat_Brain_SAL10.2_PDE2_CA1_2_Merge.oif	94.24325012207045
201955	Rat_Brain_SAL10.2_PDE2_CA1_2_Merge.oif	123.94103203667554
201955	Rat_Brain_SAL10.2_PDE2_CA1_2_Merge.oif	59.7388907877605
201955	Rat_Brain_SAL10.2_PDE2_CA1_2_Merge.oif	98.5348371039498
201955	Rat_Brain_SAL10.2_PDE2_CA1_2_Merge.oif	141.45070692274328
201955	Rat_Brain_SAL10.2_PDE2_CA1_2_Merge.oif	93.89992316352011
201955	Rat_Brain_SAL10.2_PDE2_CA1_2_Merge.oif	94.92990403917115
201955	Rat_Brain_SAL10.2_PDE2_CA1_2_Merge.oif	398.9459258355041
201955	Rat_Brain_SAL10.2_PDE2_CA1_2_Merge.oif	85.31674919976142
201955	Rat_Brain_SAL10.2_PDE2_CA1_2_Merge.oif	38.795946316189294
201955	Rat_Brain_SAL10.2_PDE2_CA1_2_Merge.oif	207.5411464436852
201955	Rat_Brain_SAL10.2_PDE2_CA1_2_Merge.oif	123.25437811957484
201955	Rat_Brain_SAL10.2_PDE2_CA1_2_Merge.oif	376.80133700900666
201955	Rat_Brain_SAL10.2_PDE2_CA1_2_Merge.oif	205.99617513020866
201955	Rat_Brain_SAL10.2_PDE2_CA1_2_Merge.oif	184.19491326226157
201955	Rat_Brain_SAL10.2_PDE2_CA1_2_Merge.oif	69.18038214789507
201955	Rat_Brain_SAL10.2_PDE2_CA1_2_Merge.oif	435.5102469211161
201955	Rat_Brain_SAL10.2_PDE2_CA1_2_Merge.oif	117.41781982421892
201955	Rat_Brain_SAL10.2_PDE2_CA1_2_Merge.oif	133.72585035536045
201955	Rat_Brain_SAL10.2_PDE2_CA1_2_Merge.oif	161.0203435601131
201955	Rat_Brain_SAL10.2_PDE2_CA1_2_Merge.oif	234.83563964843785
201955	Rat_Brain_SAL10.2_PDE2_CA1_2_Merge.oif	199.64462639702722
201955	Rat_Brain_SAL10.2_PDE2_CA1_2_Merge.oif	483.06103068033923
201955	Rat_Brain_SAL10.2_PDE2_CA1_2_Merge.oif	71.5836708577475
201955	Rat_Brain_SAL10.2_PDE2_CA1_2_Merge.oif	98.19151014539946
201955	Rat_Brain_SAL10.2_PDE2_CA1_2_Merge.oif	88.40669182671454
201955	Rat_Brain_SAL10.2_PDE2_CA1_2_Merge.oif	306.9343009440109
201955	Rat_Brain_SAL10.2_PDE2_CA1_2_Merge.oif	63.68715081108951
201955	Rat_Brain_SAL10.2_PDE2_CA1_2_Merge.oif	109.00630933973541
201955	Rat_Brain_SAL10.2_PDE2_CA1_2_Merge.oif	35.36267673068582
201955	Rat_Brain_SAL10.2_PDE2_CA1_2_Merge.oif	125.82933030870245

201955	Rat_Brain_SAL10.2_PDE2_CA1_2_Merge.oif	238.61223619249168
201955	Rat_Brain_SAL10.2_PDE2_CA1_2_Merge.oif	115.87284851074237
201955	Rat_Brain_SAL10.2_PDE2_CA1_2_Merge.oif	195.52470289442303
201955	Rat_Brain_SAL10.2_PDE2_CA1_2_Merge.oif	193.29307766384576
201955	Rat_Brain_SAL10.2_PDE2_CA1_2_Merge.oif	180.5899801974829
201955	Rat_Brain_SAL10.2_PDE2_CA1_2_Merge.oif	85.31674919976142
201955	Rat_Brain_SAL10.2_PDE2_CA1_2_Merge.oif	44.97583157009556
201955	Rat_Brain_SAL10.2_PDE2_CA1_2_Merge.oif	362.0382777913417
201955	Rat_Brain_SAL10.2_PDE2_CA1_2_Merge.oif	204.10787685818173
201955	Rat_Brain_SAL10.2_PDE2_CA1_2_Merge.oif	56.820611640082554
201955	Rat_Brain_SAL10.2_PDE2_CA1_2_Merge.oif	79.82351786295585
201956	Rat_Brain_SAL15.2_PDE2_CA1_Merge.oif	21.457934909396734
201956	Rat_Brain_SAL15.2_PDE2_CA1_Merge.oif	114.84286763509131
201956	Rat_Brain_SAL15.2_PDE2_CA1_Merge.oif	55.79063076443151
201956	Rat_Brain_SAL15.2_PDE2_CA1_Merge.oif	67.29208387586816
201956	Rat_Brain_SAL15.2_PDE2_CA1_Merge.oif	83.94344136556003
201956	Rat_Brain_SAL15.2_PDE2_CA1_Merge.oif	138.70409125434048
201956	Rat_Brain_SAL15.2_PDE2_CA1_Merge.oif	47.894110717773515
201956	Rat_Brain_SAL15.2_PDE2_CA1_Merge.oif	44.117514173719684
201956	Rat_Brain_SAL15.2_PDE2_CA1_Merge.oif	87.72003790961385
201956	Rat_Brain_SAL15.2_PDE2_CA1_Merge.oif	247.7104005940759
201956	Rat_Brain_SAL15.2_PDE2_CA1_Merge.oif	47.37912027994799
201956	Rat_Brain_SAL15.2_PDE2_CA1_Merge.oif	102.4830971272788
201956	Rat_Brain_SAL15.2_PDE2_CA1_Merge.oif	208.74279079861142
201956	Rat_Brain_SAL15.2_PDE2_CA1_Merge.oif	103.1697510443795
201956	Rat_Brain_SAL15.2_PDE2_CA1_Merge.oif	74.1586230468751
201956	Rat_Brain_SAL15.2_PDE2_CA1_Merge.oif	198.27131856282583
201956	Rat_Brain_SAL15.2_PDE2_CA1_Merge.oif	87.03338399251315
201956	Rat_Brain_SAL15.2_PDE2_CA1_Merge.oif	209.25778123643695
201956	Rat_Brain_SAL15.2_PDE2_CA1_Merge.oif	124.1126955159507
201956	Rat_Brain_SAL15.2_PDE2_CA1_Merge.oif	29.86944539388025
201956	Rat_Brain_SAL15.2_PDE2_CA1_Merge.oif	189.8598080783423
201956	Rat_Brain_SAL15.2_PDE2_CA1_Merge.oif	120.8510894097224
201956	Rat_Brain_SAL15.2_PDE2_CA1_Merge.oif	43.60252373589416
201956	Rat_Brain_SAL15.2_PDE2_CA1_Merge.oif	45.834148966471425
201956	Rat_Brain_SAL15.2_PDE2_CA1_Merge.oif	102.4830971272788
201956	Rat_Brain_SAL15.2_PDE2_CA1_Merge.oif	149.6905539279516
201956	Rat_Brain_SAL15.2_PDE2_CA1_Merge.oif	95.78822143554703
201956	Rat_Brain_SAL15.2_PDE2_CA1_Merge.oif	255.0919302029084
201956	Rat_Brain_SAL15.2_PDE2_CA1_Merge.oif	90.98164401584216
201956	Rat_Brain_SAL15.2_PDE2_CA1_Merge.oif	18.539655761718777
201956	Rat_Brain_SAL15.2_PDE2_CA1_Merge.oif	62.31384297688811
201956	Rat_Brain_SAL15.2_PDE2_CA1_Merge.oif	168.40187316894557
201956	Rat_Brain_SAL15.2_PDE2_CA1_Merge.oif	172.69346015082493
201956	Rat_Brain_SAL15.2_PDE2_CA1_Merge.oif	88.40669182671454
201956	Rat_Brain_SAL15.2_PDE2_CA1_Merge.oif	20.084627075195346
201956	Rat_Brain_SAL15.2_PDE2_CA1_Merge.oif	126.51598422580314
201956	Rat_Brain_SAL15.2_PDE2_CA1_Merge.oif	71.92699781629786
201956	Rat_Brain_SAL15.2_PDE2_CA1_Merge.oif	111.0662710910375
201956	Rat_Brain_SAL15.2_PDE2_CA1_Merge.oif	215.60932996961836

201956	Rat_Brain_SAL15.2_PDE2_CA1_Merge.oif	254.2336128065325
201956	Rat_Brain_SAL15.2_PDE2_CA1_Merge.oif	83.94344136556003
201956	Rat_Brain_SAL15.2_PDE2_CA1_Merge.oif	38.1092923990886
201956	Rat_Brain_SAL15.2_PDE2_CA1_Merge.oif	215.60932996961836
201956	Rat_Brain_SAL15.2_PDE2_CA1_Merge.oif	229.68573527018262
201956	Rat_Brain_SAL15.2_PDE2_CA1_Merge.oif	241.35885186089448
201956	Rat_Brain_SAL15.2_PDE2_CA1_Merge.oif	71.24034389919716
201956	Rat_Brain_SAL15.2_PDE2_CA1_Merge.oif	200.67460727267826
201956	Rat_Brain_SAL15.2_PDE2_CA1_Merge.oif	146.08562086317295
201956	Rat_Brain_SAL15.2_PDE2_CA1_Merge.oif	10.299808756510432
201956	Rat_Brain_SAL15.2_PDE2_CA1_Merge.oif	207.19781948513486
201956	Rat_Brain_SAL15.2_PDE2_CA1_Merge.oif	67.63541083441851
201956	Rat_Brain_SAL15.2_PDE2_CA1_Merge.oif	112.95456936306442
201956	Rat_Brain_SAL15.2_PDE2_CA1_Merge.oif	112.61124240451406
201956	Rat_Brain_SAL15.2_PDE2_CA1_Merge.oif	56.820611640082554
201956	Rat_Brain_SAL15.2_PDE2_CA1_Merge.oif	64.88879516601573
201956	Rat_Brain_SAL15.2_PDE2_CA1_Merge.oif	49.09575507269973
201956	Rat_Brain_SAL15.2_PDE2_CA1_Merge.oif	27.98114712185334
201956	Rat_Brain_SAL15.2_PDE2_CA1_Merge.oif	176.1267297363284
201956	Rat_Brain_SAL15.2_PDE2_CA1_Merge.oif	93.89992316352011
201956	Rat_Brain_SAL15.2_PDE2_CA1_Merge.oif	197.0696742078996
201956	Rat_Brain_SAL15.2_PDE2_CA1_Merge.oif	52.35736117892803
201956	Rat_Brain_SAL15.2_PDE2_CA1_Merge.oif	115.01453111436649
201956	Rat_Brain_SAL15.2_PDE2_CA1_Merge.oif	116.55950242784306
201956	Rat_Brain_SAL15.2_PDE2_CA1_Merge.oif	167.02856533474417
201956	Rat_Brain_SAL15.2_PDE2_CA1_Merge.oif	41.19923502604173
201956	Rat_Brain_SAL15.2_PDE2_CA1_Merge.oif	255.26359368218354
201956	Rat_Brain_SAL15.2_PDE2_CA1_Merge.oif	213.3777047390411
201956	Rat_Brain_SAL15.2_PDE2_CA1_Merge.oif	80.85349873860689
201956	Rat_Brain_SAL15.2_PDE2_CA1_Merge.oif	46.00581244574659
201956	Rat_Brain_SAL15.2_PDE2_CA1_Merge.oif	91.15330749511733
201956	Rat_Brain_SAL15.2_PDE2_CA1_Merge.oif	96.13154839409736
201956	Rat_Brain_SAL15.2_PDE2_CA1_Merge.oif	116.04451199001754
201956	Rat_Brain_SAL15.2_PDE2_CA1_Merge.oif	146.4289478217233
201956	Rat_Brain_SAL15.2_PDE2_CA1_Merge.oif	95.61655795627185
201956	Rat_Brain_SAL15.2_PDE2_CA1_Merge.oif	45.834148966471425
201956	Rat_Brain_SAL15.2_PDE2_CA1_Merge.oif	84.80175876193589
201956	Rat_Brain_SAL15.2_PDE2_CA1_Merge.oif	73.30030565049925
201956	Rat_Brain_SAL15.2_PDE2_CA1_Merge.oif	104.71472235785606
201956	Rat_Brain_SAL15.2_PDE2_CA1_Merge.oif	283.2447408040369
201956	Rat_Brain_SAL15.2_PDE2_CA1_Merge.oif	114.84286763509131
201956	Rat_Brain_SAL15.2_PDE2_CA1_Merge.oif	24.03288709852434
201956	Rat_Brain_SAL15.2_PDE2_CA1_Merge.oif	73.98695956759994
201957	Rat_Brain_SAL1.3_PDE2_CA1_Merge.oif	115.87284851074237
201957	Rat_Brain_SAL1.3_PDE2_CA1_Merge.oif	105.40137627495676
201957	Rat_Brain_SAL1.3_PDE2_CA1_Merge.oif	31.071089748806468
201957	Rat_Brain_SAL1.3_PDE2_CA1_Merge.oif	379.20462571885906
201957	Rat_Brain_SAL1.3_PDE2_CA1_Merge.oif	404.78248413086
201957	Rat_Brain_SAL1.3_PDE2_CA1_Merge.oif	456.10986443413697
201957	Rat_Brain_SAL1.3_PDE2_CA1_Merge.oif	193.29307766384576

201957	Rat_Brain_SAL1.3_PDE2_CA1_Merge.oif	417.48558159722285
201957	Rat_Brain_SAL1.3_PDE2_CA1_Merge.oif	114.84286763509131
201957	Rat_Brain_SAL1.3_PDE2_CA1_Merge.oif	415.4256198459208
201957	Rat_Brain_SAL1.3_PDE2_CA1_Merge.oif	436.368564317492
201957	Rat_Brain_SAL1.3_PDE2_CA1_Merge.oif	105.74470323350711
201957	Rat_Brain_SAL1.3_PDE2_CA1_Merge.oif	609.4053514268672
201957	Rat_Brain_SAL1.3_PDE2_CA1_Merge.oif	410.4473789469407
201957	Rat_Brain_SAL1.3_PDE2_CA1_Merge.oif	1233.058771633574
201957	Rat_Brain_SAL1.3_PDE2_CA1_Merge.oif	622.6234393310556
201957	Rat_Brain_SAL1.3_PDE2_CA1_Merge.oif	893.336746148005
201957	Rat_Brain_SAL1.3_PDE2_CA1_Merge.oif	70.03869954427094
201957	Rat_Brain_SAL1.3_PDE2_CA1_Merge.oif	516.363745659723
201957	Rat_Brain_SAL1.3_PDE2_CA1_Merge.oif	221.10256130642398
201957	Rat_Brain_SAL1.3_PDE2_CA1_Merge.oif	927.4977785237644
201957	Rat_Brain_SAL1.3_PDE2_CA1_Merge.oif	224.19250393337708
201957	Rat_Brain_SAL1.3_PDE2_CA1_Merge.oif	668.9725787353526
201957	Rat_Brain_SAL1.3_PDE2_CA1_Merge.oif	13.046424424913214
201957	Rat_Brain_SAL1.3_PDE2_CA1_Merge.oif	162.05032443576414
201957	Rat_Brain_SAL1.3_PDE2_CA1_Merge.oif	470.1862697347012
201957	Rat_Brain_SAL1.3_PDE2_CA1_Merge.oif	266.59338331434503
201957	Rat_Brain_SAL1.3_PDE2_CA1_Merge.oif	470.70126017252676
201957	Rat_Brain_SAL1.3_PDE2_CA1_Merge.oif	431.2186599392367
201957	Rat_Brain_SAL1.3_PDE2_CA1_Merge.oif	67.12042039659299
201957	Rat_Brain_SAL1.3_PDE2_CA1_Merge.oif	330.10887064615935
201958	Rat_Brain_SAL6DX_PDE2_CA1_Merge_2.oif	251.4869971381297
201958	Rat_Brain_SAL6DX_PDE2_CA1_Merge_2.oif	22.48791578504778
201958	Rat_Brain_SAL6DX_PDE2_CA1_Merge_2.oif	638.0731524658213
201958	Rat_Brain_SAL6DX_PDE2_CA1_Merge_2.oif	12.703097466362868
201958	Rat_Brain_SAL6DX_PDE2_CA1_Merge_2.oif	221.61755174424945
201958	Rat_Brain_SAL6DX_PDE2_CA1_Merge_2.oif	1028.779231296117
201958	Rat_Brain_SAL6DX_PDE2_CA1_Merge_2.oif	174.9250853814022
201958	Rat_Brain_SAL6DX_PDE2_CA1_Merge_2.oif	626.2283723958343
201958	Rat_Brain_SAL6DX_PDE2_CA1_Merge_2.oif	1247.4785038926887
201958	Rat_Brain_SAL6DX_PDE2_CA1_Merge_2.oif	215.78099344889355
201958	Rat_Brain_SAL6DX_PDE2_CA1_Merge_2.oif	1041.3106652832048
201958	Rat_Brain_SAL6DX_PDE2_CA1_Merge_2.oif	278.95315382215756
201958	Rat_Brain_SAL6DX_PDE2_CA1_Merge_2.oif	682.5339935980913
201958	Rat_Brain_SAL6DX_PDE2_CA1_Merge_2.oif	82.22680657280829
201958	Rat_Brain_SAL6DX_PDE2_CA1_Merge_2.oif	371.3081056722011
201958	Rat_Brain_SAL6DX_PDE2_CA1_Merge_2.oif	262.1301328531905
201958	Rat_Brain_SAL6DX_PDE2_CA1_Merge_2.oif	248.3970545111766
201958	Rat_Brain_SAL6DX_PDE2_CA1_Merge_2.oif	436.71189127604237
201958	Rat_Brain_SAL6DX_PDE2_CA1_Merge_2.oif	70.72535346137164
201958	Rat_Brain_SAL6DX_PDE2_CA1_Merge_2.oif	319.2940714518234
201958	Rat_Brain_SAL6DX_PDE2_CA1_Merge_2.oif	796.3468803575316
201958	Rat_Brain_SAL6DX_PDE2_CA1_Merge_2.oif	241.53051534016961
201958	Rat_Brain_SAL6DX_PDE2_CA1_Merge_2.oif	499.54072469075595
201958	Rat_Brain_SAL6DX_PDE2_CA1_Merge_2.oif	136.30080254448805
201958	Rat_Brain_SAL6DX_PDE2_CA1_Merge_2.oif	620.5634775797536
201958	Rat_Brain_SAL6DX_PDE2_CA1_Merge_2.oif	409.76072502984005

201958	Rat_Brain_SAL6DX_PDE2_CA1_Merge_2.oif	480.3144150119365
201958	Rat_Brain_SAL6DX_PDE2_CA1_Merge_2.oif	568.721106838651
201958	Rat_Brain_SAL6DX_PDE2_CA1_Merge_2.oif	751.1993853081609
201958	Rat_Brain_SAL6DX_PDE2_CA1_Merge_2.oif	185.73988457573813
201958	Rat_Brain_SAL6DX_PDE2_CA1_Merge_2.oif	259.21185370551257
201958	Rat_Brain_SAL6DX_PDE2_CA1_Merge_2.oif	113.64122328016511
201958	Rat_Brain_SAL6DX_PDE2_CA1_Merge_2.oif	235.69395704481371
201958	Rat_Brain_SAL6DX_PDE2_CA1_Merge_2.oif	575.587646009658
201958	Rat_Brain_SAL6DX_PDE2_CA1_Merge_2.oif	17.16634792751739
201958	Rat_Brain_SAL6DX_PDE2_CA1_Merge_2.oif	17.509674886067735
201959	Rat_Brain_SAL15.1_PDE2_CA1_2_Merge_2.oif	148.14558261447505
201959	Rat_Brain_SAL15.1_PDE2_CA1_2_Merge_2.oif	261.9584693739153
201959	Rat_Brain_SAL15.1_PDE2_CA1_2_Merge_2.oif	17.68133836534291
201959	Rat_Brain_SAL15.1_PDE2_CA1_2_Merge_2.oif	192.94975070529543
201959	Rat_Brain_SAL15.1_PDE2_CA1_2_Merge_2.oif	117.58948330349412
201959	Rat_Brain_SAL15.1_PDE2_CA1_2_Merge_2.oif	230.5440526665585
201959	Rat_Brain_SAL15.1_PDE2_CA1_2_Merge_2.oif	278.43816338433203
201959	Rat_Brain_SAL15.1_PDE2_CA1_2_Merge_2.oif	241.18718838161928
201959	Rat_Brain_SAL15.1_PDE2_CA1_2_Merge_2.oif	86.34673007541247
201959	Rat_Brain_SAL15.1_PDE2_CA1_2_Merge_2.oif	84.97342224121107
201959	Rat_Brain_SAL15.1_PDE2_CA1_2_Merge_2.oif	104.71472235785606
201959	Rat_Brain_SAL15.1_PDE2_CA1_2_Merge_2.oif	63.00049689398881
201959	Rat_Brain_SAL15.1_PDE2_CA1_2_Merge_2.oif	255.26359368218354
201959	Rat_Brain_SAL15.1_PDE2_CA1_2_Merge_2.oif	144.19732259114608
201959	Rat_Brain_SAL15.1_PDE2_CA1_2_Merge_2.oif	95.61655795627185
201959	Rat_Brain_SAL15.1_PDE2_CA1_2_Merge_2.oif	216.12432040744392
201959	Rat_Brain_SAL15.1_PDE2_CA1_2_Merge_2.oif	33.98936889648443
201959	Rat_Brain_SAL15.1_PDE2_CA1_2_Merge_2.oif	162.39365139431447
201959	Rat_Brain_SAL15.1_PDE2_CA1_2_Merge_2.oif	57.507265557183246
201959	Rat_Brain_SAL15.1_PDE2_CA1_2_Merge_2.oif	89.26500922309042
201959	Rat_Brain_SAL15.1_PDE2_CA1_2_Merge_2.oif	132.52420600043425
201959	Rat_Brain_SAL15.1_PDE2_CA1_2_Merge_2.oif	107.1180110677085
201959	Rat_Brain_SAL15.1_PDE2_CA1_2_Merge_2.oif	161.87866095648897
201959	Rat_Brain_SAL15.1_PDE2_CA1_2_Merge_2.oif	169.08852708604627
201959	Rat_Brain_SAL15.1_PDE2_CA1_2_Merge_2.oif	48.06577419704868
201959	Rat_Brain_SAL15.1_PDE2_CA1_2_Merge_2.oif	35.534340209960995
201959	Rat_Brain_SAL15.1_PDE2_CA1_2_Merge_2.oif	243.59047709147174
201959	Rat_Brain_SAL15.1_PDE2_CA1_2_Merge_2.oif	118.44780069986997
201959	Rat_Brain_SAL15.1_PDE2_CA1_2_Merge_2.oif	158.61705485026067
201959	Rat_Brain_SAL15.1_PDE2_CA1_2_Merge_2.oif	185.2248941379126
201959	Rat_Brain_SAL15.1_PDE2_CA1_2_Merge_2.oif	147.63059217664954
201959	Rat_Brain_SAL15.1_PDE2_CA1_2_Merge_2.oif	142.30902431911915
201959	Rat_Brain_SAL15.1_PDE2_CA1_2_Merge_2.oif	20.256290554470517
201959	Rat_Brain_SAL15.1_PDE2_CA1_2_Merge_2.oif	125.14267639160177
201959	Rat_Brain_SAL15.1_PDE2_CA1_2_Merge_2.oif	167.3718922932945
201959	Rat_Brain_SAL15.1_PDE2_CA1_2_Merge_2.oif	92.18328837076838
201959	Rat_Brain_SAL15.1_PDE2_CA1_2_Merge_2.oif	190.546461995443
201959	Rat_Brain_SAL15.1_PDE2_CA1_2_Merge_2.oif	148.83223653157575
201959	Rat_Brain_SAL15.1_PDE2_CA1_2_Merge_2.oif	118.61946417914515
201959	Rat_Brain_SAL15.1_PDE2_CA1_2_Merge_2.oif	48.75242811414938

201959	Rat_Brain_SAL15.1_PDE2_CA1_2_Merge_2.oif	82.0551430935331
201959	Rat_Brain_SAL15.1_PDE2_CA1_2_Merge_2.oif	280.4981251356341
201959	Rat_Brain_SAL15.1_PDE2_CA1_2_Merge_2.oif	123.08271464029967
201959	Rat_Brain_SAL15.1_PDE2_CA1_2_Merge_2.oif	418.1722355143236
201959	Rat_Brain_SAL15.1_PDE2_CA1_2_Merge_2.oif	58.53724643283429
201959	Rat_Brain_SAL15.1_PDE2_CA1_2_Merge_2.oif	30.899426269531297
201959	Rat_Brain_SAL15.1_PDE2_CA1_2_Merge_2.oif	322.38401407877654
201959	Rat_Brain_SAL15.1_PDE2_CA1_2_Merge_2.oif	120.67942593044724
201959	Rat_Brain_SAL15.1_PDE2_CA1_2_Merge_2.oif	66.94875691731781
201959	Rat_Brain_SAL15.1_PDE2_CA1_2_Merge_2.oif	216.12432040744392
201959	Rat_Brain_SAL15.1_PDE2_CA1_2_Merge_2.oif	206.85449252658452
201959	Rat_Brain_SAL15.1_PDE2_CA1_2_Merge_2.oif	223.50585001627638
201959	Rat_Brain_SAL15.1_PDE2_CA1_2_Merge_2.oif	62.65716993543847
201959	Rat_Brain_SAL15.1_PDE2_CA1_2_Merge_2.oif	238.61223619249168
201959	Rat_Brain_SAL15.1_PDE2_CA1_2_Merge_2.oif	182.99326890733536
201959	Rat_Brain_SAL15.1_PDE2_CA1_2_Merge_2.oif	168.7452001274959
201959	Rat_Brain_SAL15.1_PDE2_CA1_2_Merge_2.oif	63.68715081108951
201959	Rat_Brain_SAL15.1_PDE2_CA1_2_Merge_2.oif	134.75583123101148
201959	Rat_Brain_SAL15.1_PDE2_CA1_2_Merge_2.oif	92.6982788085939
201959	Rat_Brain_SAL15.1_PDE2_CA1_2_Merge_2.oif	104.71472235785606
201959	Rat_Brain_SAL15.1_PDE2_CA1_2_Merge_2.oif	361.86661431206653
201959	Rat_Brain_SAL15.1_PDE2_CA1_2_Merge_2.oif	111.0662710910375
201959	Rat_Brain_SAL15.1_PDE2_CA1_2_Merge_2.oif	25.921185370551253
201959	Rat_Brain_SAL15.1_PDE2_CA1_2_Merge_2.oif	279.2964807807079
201959	Rat_Brain_SAL15.1_PDE2_CA1_2_Merge_2.oif	170.97682535807317
201959	Rat_Brain_SAL15.1_PDE2_CA1_2_Merge_2.oif	171.83514275444904
201959	Rat_Brain_SAL15.1_PDE2_CA1_2_Merge_2.oif	42.91586981879347
201959	Rat_Brain_SAL15.1_PDE2_CA1_2_Merge_2.oif	56.64894816080738
201959	Rat_Brain_SAL15.1_PDE2_CA1_2_Merge_2.oif	156.21376614040824
201959	Rat_Brain_SAL15.1_PDE2_CA1_2_Merge_2.oif	187.79984632704023
201959	Rat_Brain_SAL15.1_PDE2_CA1_2_Merge_2.oif	55.79063076443151
201959	Rat_Brain_SAL15.1_PDE2_CA1_2_Merge_2.oif	186.7698654513892
201959	Rat_Brain_SAL15.1_PDE2_CA1_2_Merge_2.oif	67.97873779296886
201959	Rat_Brain_SAL15.1_PDE2_CA1_2_Merge_2.oif	198.44298204210102
201959	Rat_Brain_SAL15.1_PDE2_CA1_2_Merge_2.oif	197.2413376871748
201959	Rat_Brain_SAL15.1_PDE2_CA1_2_Merge_2.oif	27.80948364257817
201959	Rat_Brain_SAL15.1_PDE2_CA1_2_Merge_2.oif	323.2423314751524
201959	Rat_Brain_SAL15.1_PDE2_CA1_2_Merge_2.oif	147.45892869737435
201959	Rat_Brain_SAL15.1_PDE2_CA1_2_Merge_2.oif	173.20845058865044
201959	Rat_Brain_SAL15.1_PDE2_CA1_2_Merge_2.oif	142.4806877983943
201959	Rat_Brain_SAL15.1_PDE2_CA1_2_Merge_2.oif	370.9647787136508
201959	Rat_Brain_SAL15.1_PDE2_CA1_2_Merge_2.oif	335.9454289415153
201959	Rat_Brain_SAL15.1_PDE2_CA1_2_Merge_2.oif	254.74860324435804
201959	Rat_Brain_SAL15.1_PDE2_CA1_2_Merge_2.oif	131.32256164550802
201959	Rat_Brain_SAL15.1_PDE2_CA1_2_Merge_2.oif	19.397973158094647
201959	Rat_Brain_SAL15.1_PDE2_CA1_2_Merge_2.oif	242.04550577799517
201959	Rat_Brain_SAL15.1_PDE2_CA1_2_Merge_2.oif	266.7650467936202
201959	Rat_Brain_SAL15.1_PDE2_CA1_2_Merge_2.oif	179.2166723632815
201959	Rat_Brain_SAL15.1_PDE2_CA1_2_Merge_2.oif	86.0034031168621
201959	Rat_Brain_SAL15.1_PDE2_CA1_2_Merge_2.oif	30.384435831705776

of SAL-treated rats

Mean
521.0366088631985
464.1598440545809
564.0
497.89035667107004
425.5054229934924
408.3160721135405
514.0006583278473
459.4268366727383
617.1837387964149
562.5279449699054
716.8896103896104
512.6090190609019
618.9374348279458
651.9847250509165
575.8282591725215
244.35
533.777331995988
565.3076923076923
557.6140845070422
587.3735070575461
575.7667984189724
628.8711217183771
633.4297782470961
656.4003062787136
722.2565597667639
685.5638766519824
670.9036144578313
551.9969834087481
616.3586723768736
564.9069579288026
524.9636363636364
538.7232432432432
948.0892857142857
682.1491569390402
640.8328748280605
656.1736281736281
680.3293250581846
797.0272108843537
625.9293617021276
676.0398613518198
800.2331932773109
629.6173913043478
643.8567298425955
833.7377049180328
677.3207008964955
716.2708333333334
634.5973871733967
656.5597738287561

520.2535046728972
499.5589098532495
512.0358072916666
521.087069525666
520.1454743333938
461.9709122961657
568.7833333333333
500.4160789844852
391.0088495575221
476.3474240422721
406.48524404086265
448.17322097378275
505.76654314452094
373.2875
516.1321668909825
418.53246753246754
569.8038942976356
660.6553524804177
715.5441767068273
739.920731707317
648.2609170305677
611.5692307692308
653.7553041018388
680.7526636225266
658.6246648793566
539.3389626055489
693.3331911262799
462.01413427561835
589.8042604501608
445.4091209517515
750.5201863354038
634.2673869007427
554.0802469135803
859.5263157894736
781.2264150943396
476.67907801418437
1029.4906832298136
791.0932038834951
435.352526439483
430.7966804979253
569.1607262945528
621.7023121387283
602.6495652173913
476.9967447916667
552.6
782.8032564450475
862.7933425797504
728.0783847980998
882.0227552275522
869.3661971830986

1218.689393939394
794.4506387921022
1071.2062350119904
743.5430463576159
735.0924024640657
1397.580027359781
748.3358925143954
695.3870967741935
691.6540983606558
1077.7980358667805
553.1358024691358
863.6263157894737
1025.5341938405797
854.358844765343
829.7159309021113
673.7137745974956
751.2734246575343
606.016393442623
601.5145482388974
582.0382513661202
643.9279778393352
624.9971264367816
1125.7996515679442
679.5036407766991
943.8793418647166
549.7721518987341
676.2078313253012
552.5191146881288
996.5840707964602
806.2258064516129
679.8008356545961
809.3002277904328
806.5908333333333
793.2777260018639
570.5657568238213
872.7654710287742
1305.3157894736842
927.1078305519898
849.7366737739873
944.2631578947369
1128.4075666380052
958.9804548685146
709.0623501199041
753.9615384615385
824.147572815534
812.9099552572707
844.9973045822103
702.0629921259842
742.9514563106796
1167.9495225102319

772.9532374100719
755.1481481481482
592.3538191395961
662.6936056838366
607.4857414448669
541.1448692152918
693.087786259542
646.348506401138
803.6846089150547
972.1601208459215
690.4774193548387
575.784
513.9641255605382
496.5230769230769
457.2908163265306
460.42535787321066
431.81064356435644
461.39426523297493
482.2023346303502
530.8043052837573
513.2245322245323
431.3659420289855
505.8643216080402
563.3659539473684
497.71214642262896
485.5324074074074
489.7099567099567
490.55029585798815
453.2149302707137
509.23236514522824
454.2068965517241
425.50542495479203
428.14914772727275
483.4251968503937
381.3632958801498
425.8676716917923
448.3520642201835
437.0358422939068
466.56325706594885
429.46792452830186
472.6111111111111
571.068870523416
459.32415902140673
479.86580516898607
457.5747572815534
529.3504273504274
467.4369063772049
430.14081145584726
522.2720247295209
462.1656050955414

462.2261985145172
512.5664621676891
512.7162162162163
383.8423566878981
471.4723467862481
539.645092460882
456.6481927710843
499.94781864841747
490.54289071680375
489.98333333333335
467.52361226180614
462.2690355329949
477.98480243161094
515.9298780487804
531.2749244712991
478.962962962963
534.6993006993007
588.9815950920246
507.89863547758284
547.2084095063985
538.0601045296168
517.8950819672131
468.3731343283582
513.338733431517
524.5981500513875
550.825
548.5669132481506
520.8037007240547
511.4564755838641
435.32835820895525
495.1732580037665
453.6982142857143
452.11686390532543
514.6271981242672
546.6840215439856
417.65168539325845
539.5465587044534
515.7775175644028
443.1393442622951
497.0969696969697
543.50822122571
444.97857142857146
557.030162412993
316.97185185185185
323.5684039087948
314.4696132596685
330.93435943866
338.61747243426635
352.1309747835905
350.59591474245116

345.26356907894734
343.0179372197309
346.5615702479339
350.60818253343825
335.02597402597405
344.2014084507042
376.29150982852366
365.3758875121815
362.29114971050456
393.05745580322827
293.99754901960785
379.00531914893617
406.3167701863354
352.6439015361836
397.93950995405817
368.96150885296385
338.88157894736844
329.21292372881356
411.11865644395766
330.65679330328396
372.0733041575492
344.02109872611464
368.7749360613811
338.4316172646906
421.13924914675766
403.7022900763359
421.924670433145
333.9864864864865
314.02013942680094
357.8393125312865
376.6751717369971
362.7894736842105
370.25608917022157
387.86634844868735
392.22354104846687
367.4781538461539
354.4610160965795
345.24425887265136
364.20388349514565
389.4479371316306
341.84588804422947
389.52240566037733
402.38106796116506
417.77204301075267
415.9325285621901
474.1279317697228
395.2017182130584
482.9886649874055
325.0390041493776
424.5186426476749

423.11222301644034
413.9209175973438
391.82655393053017
418.7181146025878
423.33046357615893
393.4441087613293
429.6453022578296
445.6322696093051
276.08
352.9901960784314
1083.018539976825
1020.6710353866317
1184.504854368932
820.1298932384342
1013.1897810218978
979.0044676098288
902.2348951911221
845.373665480427
770.3996023856859
1012.0585858585858
897.6704918032787
904.3324250681198
1051.5427034297243
1116.1202380952382
750.4721723518851
903.789515488483
587.3232323232323
648.830866807611
804.7850746268657
700.7423076923077
862.9183937823834
795.3926282051282
933.9787910922588
997.6304568527919
1123.5714285714287
695.1932367149758
883.7892882311487
839.6782608695652
985.987012987013
892.8387395736793
859.4058139534884
738.0241254523522
740.4067796610169
868.2373113854595
841.3466666666667
856.6666666666666
683.9657657657658
478.5801614763553
735.5629522431259
914.8978873239437

560.4351464435147
830.6982864137087
925.1283124128313
784.4589490968801
981.5454545454545
726.4166666666666
625.4866879659212
473.3271692745377
647.1871794871795
500.8776806989674
780.7410788381743
723.5353302611367
472.3342465753425
813.2474820143885
717.922138836773
756.4506612410987
1044.1752021563343
705.5426751592356
758.15
553.5295081967213
892.4544592030361
492.03400309119013
1043.1192052980132
792.7492317148126
809.6295180722891
762.6843156843157
736.688
934.4818181818182
775.6824175824175
885.6316270566728
914.4769230769231
942.6130514705883
945.4242424242424
868.7352941176471
715.627502175805
529.3024691358024
693.3967073818375
593.7124563445867
541.017839444995
417.1674698795181
704.9014345210551
643.587123147675
655.2749326145553
772.4065359477124
479.4424778761062
513.2390070921986
592.3828828828829
658.3208812260536
714.0918163672654
589.7570621468926
